# Convergent Control of NREM Sleep and Anesthesia by Prefrontal Layer 5 Extratelencephalic Neurons

**DOI:** 10.21203/rs.3.rs-7861434/v1

**Published:** 2025-11-19

**Authors:** Andrzej Z. Wasilczuk, Daiki Takekawa, Joseph Cichon, Xiaoyan Zhang, Ethan B Blackwood, Jeffrey Parra-Munevar, Mean-Hwan Kim, William Spain, Aaron J. Krom, Max B. Kelz, Nikolai Dembrow, Alex Proekt

**Affiliations:** 1 Neuroscience of Unconsciousness and Reanimation Research Alliance, Perelman School of Medicine, University of Pennsylvania, Philadelphia, PA, USA; 2 Department of Anesthesiology and Critical Care, Perelman School of Medicine, University of Pennsylvania, Philadelphia, PA, USA; 3 Department of Anesthesiology, Graduate School of Medicine, Hirosaki University, Hirosaki, Aomori, Japan; 4 Mahoney Institute of Neurosciences, Perelman School of Medicine, University of Pennsylvania, Philadelphia, PA, USA; 5 Neuroscience Graduate Group, Perelman School of Medicine, University of Pennsylvania, Philadelphia, PA, USA; 6 Department of Neurobiology and Biophysics, University of Washington, Seattle, WA 98195, USA; 7 Synapse and Circuits Dynamics Laboratory, Department of Brain Sciences, DGIST, Daegu, South Korea; 8 Epilepsy Center of Excellence, Department of Veterans Affairs Medical Center, Seattle, WA 98108, USA; 9 Department of Anesthesiology, Intensive Care, and Pain Management, Tel Aviv Sourasky Medical Center, Tel Aviv, Israel; 10 Sagol Brain Institute, Tel Aviv Sourasky Medical Center, Tel Aviv, Israel; 11 Chronobiology and Sleep Institute, Perelman School of Medicine, University of Pennsylvania, Philadelphia, PA, USA

## Abstract

The cortical mechanisms that actively suppress consciousness remain poorly understood. Here, we identify a specific subset of prefrontal cortical (PFC) neurons preferentially active under anesthesia while most neurons are suppressed. Chemogenetic activation of these excitatory neurons enhances anesthetic potency and deepens NREM sleep, whereas their inhibition blunts anesthetic effects. We identify these NREM and Anesthesia Promoting (NAP) neurons as PFC Layer 5 extratelencephalic (L5 ET) neurons. Remarkably, NAPs have sparse cortical projections and predominately communicate with subcortical nuclei including anterior and reticular thalamic nuclei, hypothalamus, and claustrum. We identify a transgenic mouse line that labels L5 ET neurons and verify that PFC L5 ET neurons are uniquely activated under anesthesia. Furthermore, we show that activation of PFC L5 ET neurons promotes deep NREM sleep. These findings identify a unique excitatory PFC circuit that promotes both naturally-occurring and drug-induced unconsciousness, with implications for both sleep regulation and anesthetic action.

## Introduction

The state of consciousness is closely tied to thalamocortical oscillations^1^. In states of diminished consciousness such as slow wave (NREM) sleep and anesthesia, EEG exhibits slow (<4Hz) oscillations associated with rhythmic discharges of cortical and thalamic neurons^1–4^. In contrast, during wakefulness, thalamic and cortical neurons exhibit sustained irregular activity patterns associated with desynchronized EEG^1–3^. Modulation stemming from a network of mutually inhibitory arousal and sleep promoting nuclei in the brain stem, hypothalamus, and the basal forebrain^5–7^ strongly influence the oscillation patterns exhibited by the thalamocortical system.

EEG^8^ and single-neuron activity across cortex and thalamus^9^ under anesthesia closely resemble NREM sleep, indicating convergence at the level of circuit dynamics. Corroborating evidence shows that anesthetics activate sleep-promoting and suppress wake-promoting subcortical nuclei^10–17^. In addition, anesthetics directly reduce the excitability of cortical neurons^18–20^, leading to widespread suppression of cortical activity under anesthesia^21–26^. During NREM sleep, the activity of most cortical neurons is similarly reduced^27–29^. With the notable exception of heat-sensing hypothalamic^30^ and hypothalamic-projecting midbrain neurons^31^, the vast majority of all identified subcortical NREM-promoting neurons are inhibitory. Likewise, apart from habenular glutamatergic neurons^32^, the vast majority of neurons known to potentiate the effects of anesthetics are also inhibitory^10,16,17^. Recent studies suggest that the cortex also directly promotes NREM sleep^33^ and several subtypes of inhibitory cortical NREM-promoting neurons^34–38^ have been identified. Thus, suppression of consciousness appears to be primarily established through inhibition.

There are, however, compelling reasons to believe that a diminished level of consciousness may be actively brought about by excitatory neurons. For instance, NREM sleep increases following chemogenetic potentiation of excitatory synapses in the prefrontal cortex (PFC)^39–41^. Conversely, disruption of synaptic transmission in Layer 5 (L5) pyramidal neurons disrupts sleep homeostasis^42^. These results are consistent with in vivo^43,44^, cortical slab^45^, and slice work^46,47^ suggesting that slow oscillations that define states of unconsciousness originate in L5 and are critically dependent upon specific biophysical properties of L5 neurons^48^. L5 neurons, however, are a broad category composed of distinct neuronal subtypes^49,50^ with distinct properties and synaptic connectivity in different cortical regions^51–53^. The specific excitatory cortical circuits that suppress consciousness, however, have yet to be identified.

Here, using targeted recombination in active populations (TRAP)^54^, we identify a specific L5 excitatory neuron subtype in the PFC preferentially activated under anesthesia. Activation of these neurons elicits deep NREM sleep with enhanced slow-wave activity and potentiates anesthetic-induced unconsciousness, whereas their inactivation partially antagonized anesthetic EEG effects. Therefore, we refer them as NREM- and Anesthesia-Promoting (NAP) neurons. Biophysical and transcriptomic analyses classify NAPs as excitatory L5 extratelencephalic (ET) pyramidal neurons, a finding confirmed using a transgenic L5 ET mouse line^55^. Remarkably, while the PFC has extensive cortico-cortical connectivity, NAPs predominantly project to and receive inputs from subcortical nuclei known to play a key role in controlling of level of consciousness. These findings define a prefrontal circuit that actively promotes slow-wave activity in both natural and pharmacologically induced unconscious states, providing a mechanistic framework for cortical suppression of consciousness by excitatory neurons.

## Results

### Isoflurane Anesthesia Recruits a Subset of Layer 5 Prefrontal Neurons

**Figure 1:**
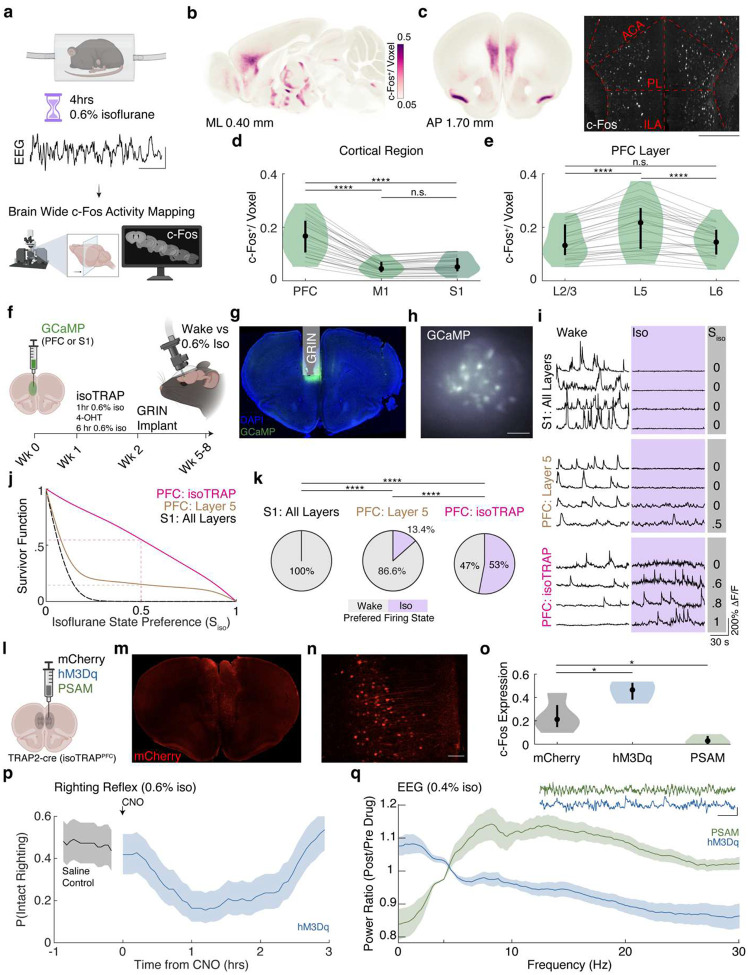
Identification of novel anesthetic active Layer 5 prefrontal cortical neurons that bidirectionally regulate anesthetic sensitivity. **a)** Experimental schematic for whole brain c-Fos activity mapping. Example EEG trace at 0.6% isoflurane. EEG is dominated by δ-oscillations. Scale bar represents 25 μV and 1 sec. **b)** Average sagittal (ML 0.4mm) and **c)** coronal (AP 1.70mm) sections showing c-Fos density (nuclei/voxel) (n=28 mice). Inset in **c)** shows representative prefrontal coronal optical section (2 μm) showing c-Fos immunoreactivity with approximate regional boundaries for anterior cingulate (ACA), prelimbic (PL), and infralimbic (ILA) cortex indicated with red lines. Scale bar represents 500μm. **d-e)** Distributions of c-Fos densities across different cortical regions and layers of the PFC (ILA, PL, ACA) shown as violin plots (gray lines show individual animals, black circle shows the median, vertical bar shows interquartile range). Mean c-Fos density (nuclei/voxel) in the prefrontal cortex (PFC) is significantly higher than primary motor (M1) or primary somatosensory (S1) cortex (F(2, 54)=166.0, p<0.0001, repeated measures 1way ANOVA; Tukey’s multiple comparison test: PFC greater than M1, p<0.0001, PFC greater than S1, p<0.0001, M1 no different from S1, p=0.48). Mean PFC density (nuclei/voxel) expression in L5 is significantly higher than L2/3 or L6 (F(2, 54) = 44.48, p<0.0001, repeated measures 1way ANOVA; Tukey’s multiple comparison test: L5 greater than L2/3, p<0.0001, L5 greater than L6, p<0.0001, L2/3 no different from L6, p=0.99). **f)** Experimental schematic for in vivo calcium imaging. Mice transfected with a GCaMP reporter were imaged either with a 2-photon microscope or 1-photon miniscope (see viral transfection and TRAP protocol in the Methods). Timeline for isoTRAP protocol is shown. **g)** Representative localization of GRIN lens in the ILA. **h)** Miniscope field of view. Scale bar represents 100 μm. **i)** Representative calcium traces obtained during wakefulness (white) and under 0.6% isoflurane (purple) in distinct neuronal populations are shown. State Preference for isoflurane (S_iso_, gray, Methods) shown for each neuron (PFC isoTRAP: n=5 mice, 83 neurons TRAPed with GCaMP under anesthesia as in **f** and recorded using miniscope; S1 All Layers: n=6 mice, 215 neurons transfected with GCaMP under synapsin promoter, and imaged with 2P microscope across all layers; PFC: Layer 5; n=4 mice, 119 cingulate neurons located more than 400 μm from pial surface, transfected with GCaMP under CamKII promoter, and imaged with a 2P microscope) **j)** Survivor function (P) of Isoflurane State Preference (S_iso_) computed for the three neuronal groups in **i**. P(0.5) reflects the fraction of neurons that fired at higher frequency under isoflurane (dashed lines). **k)** Pie charts showing the fraction of neurons that fired at higher frequency under isoflurane (purple). A chi-square test confirms that PFC isoTRAP had higher preference for firing under isoflurane (χ^2^(2)=136.7, p<0.0001 two sided. Post hoc Fisher’s exact tests (two sided) with Bonferroni correction; S1: All Layers vs PFC: Layer 5, p<0.0001; S1: All Layers vs PFC: isoTRAP, p<0.0001; PFC: Layer 5 vs PFC: isoTRAP, p<0.0001). **l)** Schematic for TRAPing PFC neurons with DREAD receptors or control inert virus using isoTRAP protocol (Methods) **m)** Validation of viral expression in the PFC. Zoom in on the ILA shown in **n** (right side corresponds to midline; scale bar is 100 μm). **o)** Validation of chemogenetic manipulations. Activation of hM3Dq-expressing neurons increases mean fraction of c-Fos expressing neurons relative to mCherry controls. PSAM-mediated inactivation reduced c-Fos expression relative to mCherry controls. (n=4 mice per group, F(2,9) = 17.67, p=0.0008, 1way ANOVA; Šídák’s multiple comparisons test: hM3Dq greater than mCherry, p=0.0291, PSAM less than mCherry, p=0.0344). **p)** Chemogenetic activation of PFC: isoTRAP neurons increases anesthetic potency as measured by decrease in righting probability (n=10 mice, mean and 95% CI estimated from Binomial distribution). **q)** Power ratios following PFC: isoTRAP chemogenetic activation (n=9 mice) or inhibition (n=6 mice) compared to pre intervention control period. Jackknife estimates across mice shown as mean and 95% CI. 30 min data analyzed before and after drug injection [30 min pre & 1–1.5hr post] for ratio calculation. Raw EEG following chemogenetic intervention is also shown. Scale Bar is 25 μV and 1 sec. Statistical significance is denoted by *p<0.05, **p<0.01, ***p<0.001, ****p<0.0001. Schematics from a, f, l were created in BioRender.

To identify neurons active under anesthesia, we performed a whole brain screen for c-Fos expression, widely used to map antecedent neuronal activity (Figure 1a)^56^. A steady state anesthetic dose was chosen to elicit consolidated slow wave activity (Figure 1a trace).^57^ Following optical clearing and immunolabeling for c-Fos,^58–60^ brains were imaged with light-sheet microscopy, enabling detection of individual c-Fos positive neurons (Figure 1a). Automated registration^61,62^ to the Allen reference atlas,^63^ allowed quantification of c-Fos expression in neurons across brain regions^63^. This screen identified numerous subcortical loci (Figure 1b), consistent with previous work showing that anesthetics activate sleep-promoting subcortical structures^11,64–67^. Surprisingly, we also observed a region of c-Fos^+^ neurons localized to the infralimbic (ILA), prelimbic (PL), and anterior cingulate (ACA) cortices — collectively referred to as the prefrontal cortex (PFC; Figure 1b–c). With the exception of piriform cortex, likely activated by the pungent odor of isoflurane,^68,69^ the rest of the neocortex had sparser c-Fos staining than the PFC (Figure 1d, PFC 0.1664 ± 0.0669, primary motor cortex (M1) 0.0506 ± 0.0257, and primary somatosensory cortex (S1) 0.0589 ± 0.0304, mean c-Fos^+^ neurons/voxel ± std). The overall fraction of PFC neurons that were c-Fos^+^ was significantly higher under isoflurane than during wakefulness (Sup. Figure 1a, Wake 0.0579 ± 0.0291, Iso 0.1081 ± 0.0405; mean ± std), suggesting that a subset of PFC neurons is preferentially activated under isoflurane anesthesia.

PFC c-Fos^+^ neurons were preferentially found in Layer 5 (L5). (Figure 1e, Layer 2/3 0.1465 ± 0.0614, L5 0.2051 ± 0.0885, and Layer 6 0.1477 ± 0.0569, mean c-Fos^+^ neurons/voxel ± std). This observation was further corroborated by c-Fos expression in Rbp4-cre x Ai6 mice, which selectively express ZsGreen fluorescent protein in L5 neurons. The number of c-Fos^+^ L5 neurons under isoflurane was approximately doubled relative to wakefulness (Sup. Figure 1b–c; Isoflurane 0.06370 ± 0.0304, Wake 0.03636 ± 0.02101, mean ± std). Thus, in contrast to most cortical neurons, a subpopulation of Layer 5 neurons in the PFC appears to be preferentially activated under anesthesia. Because, as we will show here, this specific subset of L5 PFC neurons is **N**REM sleep and **A**nesthesia **P**romoting, we hereafter refer to them as NAPs.

To obtain direct neurophysiological evidence for selective activation of NAPs under anesthesia, we used TRAP^70^ during isoflurane exposure (isoTRAP) to specifically express GCaMP in NAPs and performed in vivo calcium imaging (Figure 1f, Methods). For comparison, we also performed pan-neuronal two-photon imaging in either S1 (all layers) or in PFC Layer 5 (excitatory neurons 400 microns or deeper from cortical surface). Because most NAPs are located in the ILA—beyond the reach of conventional two-photon imaging (Figure 1c)—activity in the ILA of TRAPed mice was imaged using a miniature head-mounted microscope via GRIN lens (Figure 1 f–h).

We confirmed that NAPs were preferentially active under the anesthetized state. In contrast S1 neurons were silenced under isoflurane anesthesia (Figure 1i). To quantify the preference for activation under isoflurane, we computed a state preference index, S_iso_ (Methods). Survivor plots for S_iso_ revealed that more NAPs were preferentially active under isoflurane relative to both S1 and the excitatory L5 PFC neurons (Figure 1j). Over half of the NAPs were active at a higher rate under isoflurane (0.914 (0.3039–1.735) median transients/min (Interquartile Range)) than during wakefulness (Figure 1k). In contrast, no S1 neurons were more active under isoflurane (0%). Most PFC GABAergic interneurons (PV, SST, VIP) were also preferentially active during wakefulness (Sup. Figure 2). Consistent with the fact that NAPs are a subset of L5 neurons, small proportion of Layer 5 PFC neurons were preferentially active under isoflurane (13.4%). Thus, NAP labeling using isoTRAP achieved a ~4-fold enrichment of anesthesia-active neurons (53%) relative to L5 neurons. These results confirm that, NAPs are a subpopulation of Layer 5 PFC neurons activated under isoflurane anesthesia.

### NAPs Bidirectionally Modulate Anesthetic Potency

To test whether NAPs causally modulate anesthetic sensitivity, we selectively expressed chemogenetic modulators (hM3dq: excitatory, PSAM: inhibitory) or control mCherry virus in the PFC of TRAP2-cre mice (Figure 1l–n). c-Fos immunostaining confirmed that bidirectional chemogenetic modulation was successful in vivo (Figure 1o). Behavioral assays using repeated righting reflex revealed that hM3Dq activation of NAPs during steady state isoflurane exposure significantly increases anesthetic potency, causing more than a 50% decrease in righting probability (Figure 1p). EEG recordings under isoflurane showed that NAP activation increased the power of slow wave activity and decreased power at higher frequencies. (Figure 1q). Conversely, PSAM-mediated inactivation of NAPs produced the opposite effect on the EEG. Together, these findings demonstrate that NAP activation potentiates anesthetic effects, while their inactivation partially antagonizes anesthesia.

### NAPs are Preferentially Active During NREM Sleep

**Figure 2:**
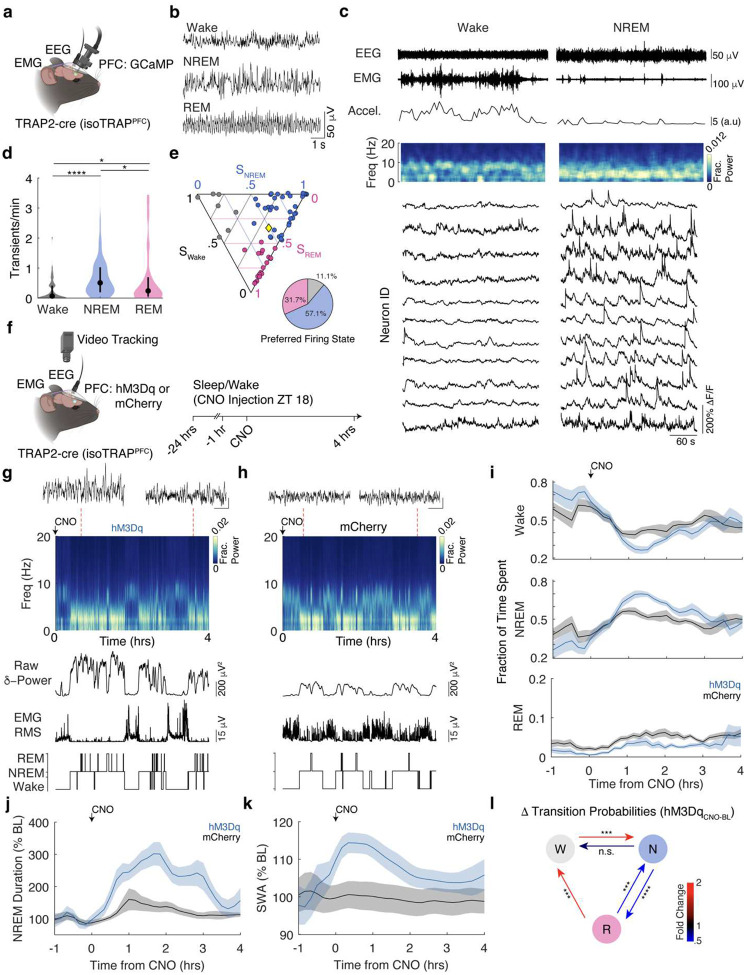
Anesthetic active PFC neurons are preferential active during NREM sleep and drive NREM sleep when chemogenetically activated. **a)** Experimental schematic for simultaneous EEG/EMG and PFC neuronal activity recordings (Methods). **b)** Representative 10 s EEG recordings classified as Wake, NREM, and REM by a neural network (Methods). **c)** 5-minute recordings of EEG, muscle tone, movement, and NAP neuronal activity from a representative mouse during wakefulness (left) and NREM sleep (right). Muscle tone and movement were computed as root mean squared (RMS) of EMG and accelerometer signals respectively. EEG spectrograms are expressed as fraction of power per frequency. **d)** Frequency of calcium transients (transients/minute) during Wake, NREM, and REM sleep shown as violin plot. Calcium transient frequency was highest in NREM sleep (n=5 mice, 63 cells, χ^2^(2)=30.28, p<0.0001, Friedman test. Post hoc Dunns multiple comparisons test: NREM>Wake, p<0.001; NREM>REM, p=0.0293; REM>Wake, p=0.0131). **e)** Distribution of state preferences shown as simplex plot (dots show individual neurons, colored according to the state in which maximal firing was observed Wake: gray, NREM; blue, or REM: pink). Yellow diamond shows mean of all neurons. Pie chart quantifies proportion of NAPs with maximal activity in each state. Distribution of neurons with state preference was significantly non-uniform (χ^2^(2) = 10.97, p<0.0041, Chi-Square Test). **f)** Experimental schematic for recording EEG/EMG with PFC isoTRAP-hM3Dq (n=11) or mCherry (n=9) mice. CNO (3mg/kg, IP) injections in both groups occurred in the middle of the dark phase (ZT 18). **g-h)** Spectrogram, delta power, EMG RMS, hypnogram, and zoomed-in EEG traces (from periods marked by red line, scale bar is 25 μV and 1 sec) from a representative hM3Dq (chemogenetic excitation) and mCherry (control) transfected mouse respectively. **i)** Fraction of time spent in each of the three states (Wake, NREM, REM) following CNO in mCherry and hM3Dq cohorts. Two way ANOVA for the fraction of time spent in NREM (F(4,90)=93.07, p<0.0001) and Wake (F(4,90)=73.75, p<0.0001) before and after CNO revealed statistically significant differences. The effect of CNO on NREM sleep depended on the mouse cohort (F(1,90)=49.83, p<0.0001, hM3Dq vs mCherry). Šídák’s multiple comparisons test revealed that hM3Dq transfected mice spent greater fraction of time in NREM sleep than the mCherry control one hour after CNO administration (13.31% [11.45 15.16], mean and 95% CI on the difference in the fraction of time spent in NREM, 1–2 hours post CNO, p<0.0001). After 3 hours following CNO administration differences in NREM sleep between the cohorts were no longer observed (0.45% [−1 2], mean and 95% CI on the difference in the fraction of time spent in NREM, 3–4 hours post CNO, p=0.6293). ANOVA performed for the fraction of time spent in REM, did not reveal significant differences (F(4,90)=1.786, p=0.1385). **j)** NREM bout duration following CNO. Two way ANOVA performed for NREM bout durations (normalized to baseline values in each cohort individually) revealed significant differences between the hM3Dq and mCherry control groups (F(4,90)=104.8, p<0.0001). Šídák’s multiple comparisons test revealed that NREM bout durations in hM3Dq cohort was longer than in mCherry controls (132.2 [121.9 142.6] percent baseline, mean 95% CI on the difference in NREM bout duration, 1–2 hours post CNO, p<0.0001). This difference decreased after 3 hours post CNO but remained statistically significant (53.58 [43.27, 63.90] percent baseline, mean 95% CI on the difference in NREM bout duration, 3–4 hours post CNO, p<0.0001). **k)** Slow-wave activity (SWA) following CNO. Two way ANOVA on SWA (normalized to pre-CNO values in each cohort individually) revealed that SWA differed significantly between hM3Dq and mCherry groups (F(4,90)=28.50, p<0.0001). Šídák’s multiple comparisons confirmed that SWA increased more in hM3Dq than in mCherry control (8.8 [7.23 10.36] mean and 95% CI on the difference between hM3Dq and mCherry 1–2 hours following CNO, p<0.0001). This difference between the cohorts diminished 3 hours after CNO administration but remained statistically significant (4.35, [2.78, 5.91], mean and 95% CI on the difference between hM3Dq and mCherry 3–4 hours following CNO, p<0.0001) **l)** Transitions between Wake, NREM, and REM were modeled as a Markov process. Transition probabilities after CNO injection (0.5–2.5 hrs) were normalized by transition probabilities zeitgeber time–matched (ZT 18.5–20.5) period 24 hrs prior to CNO injection. (n=7 hM3Dq mice, F (1.504,9.023)=97.41, p<0.0001, 2way ANOVA with Geisser-Greenhouse Correction; Šídák’s multiple comparisons test: W-N, p=0.0004; N-W, p=0.7084; N-R, p<0.0001; R-W, p=0.0003; R-N, p=0.0007. Red values indicate a greater transition probability in the CNO condition, whereas blue values indicate a greater probability in the baseline condition. Statistical significance is denoted by *p<0.05, **p<0.01, ***p<0.001, ****p<0.0001. Schematics from a, f were created in BioRender.

To test if NAPs may also participate in natural sleep, we performed calcium imaging of NAP activity in conjunction with EEG/EMG recordings in freely moving mice across naturally occurring sleep and wake states (Figure 2a) in undisturbed, home cage conditions. Vigilance states were classified as Wake, NREM, or REM (Figure 2b) using a custom neural network classifier (Sup. Figure 3). NAPs were largely silent during periods of consolidated wakefulness (Figure 2c), but displayed large, coordinated calcium transients during NREM sleep. Some NAPs exhibited increased activity during REM sleep (Sup. Figure 4). To quantify vigilance state-dependent NAP activity, we estimated calcium transient rates for each neuron in Wake, NREM and REM. Median calcium transient rates during NREM sleep were significantly higher than REM (2.1-fold difference) and wakefulness (7.5-fold difference). REM activity was higher than wakefulness (3.6-fold difference; Figure 2d; NREM: 0.5076 (0.2025–1.017); REM 0.2437 (0.0694–0.7611); Wake 0.0677 (0–0.4067); Median transients/minute (Interquartile Range)). The distribution of state preference of NAP activity, visualized as a simplex plot, was strongly non-uniform (χ^2^(2)=10.97, p=0.0041, Chi-square Test), with a strong bias toward NREM sleep (57% of NAPs were preferentially active during NREM, 32% during REM, and 11% during wakefulness, Figure 2e). Thus, NAPs activity is preferentially recruited in NREM sleep.

### NAPs Promote NREM Sleep

We next asked whether NAP activity causally contributes to sleep. NAPs were transfected with either hM3Dq or mCherry control in the PFC (Figure 2f). Vigilance states were recorded as above for 24 hours prior to CNO injection, which occurred at ZT18, the middle of the active period (Figure 2g–h). Chemogenetic activation of NAPs selectively promoted sleep over wakefulness (Figure 2i). Two-way ANOVA with Šidák’s post hoc comparisons revealed that NAP activation significantly decreased Wake time and increased NREM sleep compared to their own baseline and to the mCherry controls. Furthermore, significantly greater effect was observed in hM3Dq than in mCherry cohort for wake and NREM. REM sleep was observed following CNO injection (Sup. Figure 5), and was modestly increased in both groups. The effect of CNO on REM sleep in the hM3Dq and mCherry cohorts was not significantly different. Together, these findings indicate that NAP activation selectively promotes NREM sleep while suppressing wakefulness.

### NAPs Enhance NREM Stability

We next examined how NAP activation changes the characteristics of NREM and the dynamics of fluctuations between states of sleep and wakefulness. Chemogenetic excitation of NAPs significantly increased NREM bout duration relative to baseline (Figure 2j). Consistent with deeper, more consolidated sleep, slow-wave activity (SWA) during NREM was also significantly elevated following NAP activation (Figure 2k). In addition to electrophysiological markers, NAP activation significantly reduced locomotor activity (Sup. Figure 6) and caused decrease in body temperature consistent with natural sleep^71^ (hM3Dq Δ_temp_ −0.81 ± 0.12 °C, mCherry Δ_temp_ −0.01 ± 0.01 °C; mean ± SD; p < 0.0001, unpaired t-test), indicating a coordinated behavioral and thermoregulatory signature of enhanced NREM sleep.

To quantify the dynamics of sleep-state transitions, Wake, NREM, and REM transitions were modelled as a Markov process. Transition probability matrices were estimated 0.5–2.5 hours following CNO injection and compared to ZT-matched times from the previous day. NAP activation increased the likelihood of transitioning from Wake to NREM and reduced transitions from NREM to REM. (Figure 2l). Both of these changes in transition probabilities serve to stabilize NREM sleep. In contrast, mCherry controls showed no significant changes in transition probabilities following CNO injection (Sup. Figure 7). Together, these findings indicate that NAP activation leads to deep NREM sleep characterized by prolonged bout duration and increased slow wave activity.

### NAPs Connect with Subcortical Structures Involved in Control of Arousal and Sleep

**Figure 3:**
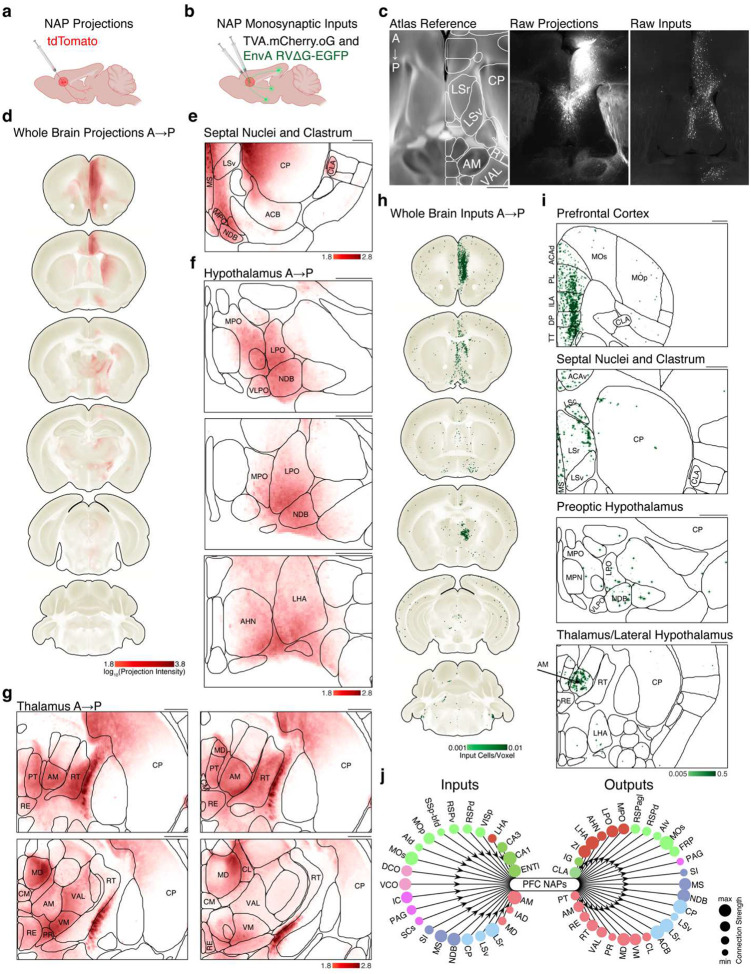
Whole brain mapping of NAP outputs and monosynaptic inputs. **a)** Experimental schematic for anterograde tracing outputs of NAPs (n=3 mice). **b)** Experimental schematic for tracing monosynaptic inputs into NAPs (n=4 mice). **c)** Transverse section through the template atlas with overlaid summary structure outlines (left), representative transverse slices (20μm maximal projection) showing anterograde projections (middle), and monosynaptic inputs (right) highlighting bidirectional communication of NAPs with anteromedial nucleus (AM) and projections to the reticular nucleus (RT). **d)** Coronal sections of the average (n=3 mice) brain-wide distribution of NAP projections **(e–g)** Region-specific insets of NAP projections to septal nuclei and claustrum **(e)**, hypothalamus **(f)**, and thalamus **(g)**. Colormap **(d-g)** shows log_10_(projection intensity per voxel). **h)** Coronal sections of the average (n=4 mice) brain-wide distribution of inputs into NAPs. **i)** Region-specific insets highlight inputs from the PFC, septal nuclei and claustrum, preoptic hypothalamus, and thalamus/lateral hypothalamus. Colormap **(h-i)** shows the density of inputs (cells/voxel) Outlines in e, f, g, and i represent boundaries of summary structures only as defined by the Allen Annotation Atlas. Fiber tracts and ventricles are not outlined. See Supplemental Table 1 for full name of brain region abbreviations and list of projection and input values for all summary structures. **j)** Connectivity plots summarizing relative strength of strongest (top 10%) inputs to (left) and outputs of (right) of NAPs. Node size encodes connection strength (scaled to the maximum value separately for inputs and outputs). Scale bars represent 500 μm across all subpanels. Schematics from a, b were created in BioRender.

The PFC is a highly heterogenous structure, projecting to and receiving inputs from diverse brain regions.^72–74^ Moreover, PFC connectivity is cell-type dependent.^53,75^ To characterize NAP synaptic connectivity, we performed brain-wide anterograde projection and monosynaptic input tracing to identify their downstream targets and upstream inputs (Figure 3a–c).

**Figure 4:**
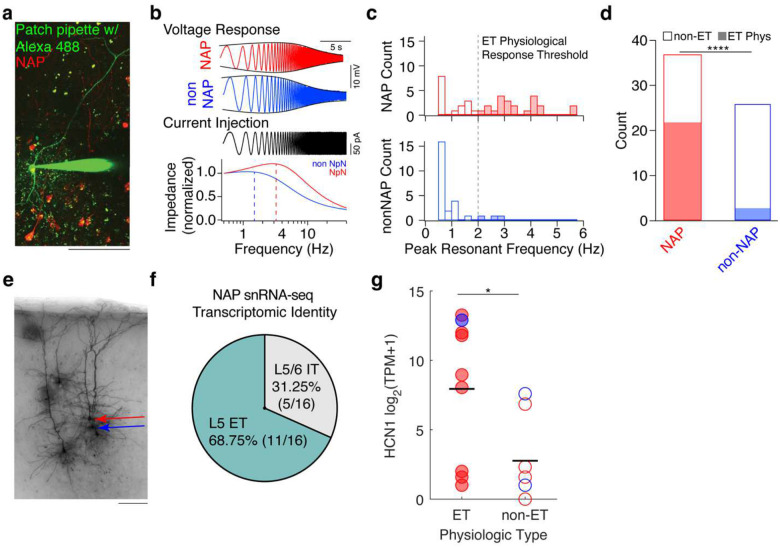
Intrinsic physiological and molecular features of NAPs. **a)** 2-photon Z-stack of NAPs (red) in medial prefrontal cortex and patch recording from a labelled L5 pyramidal neuron with Alexa 488 (green). Neurons absent of red fluorescence were considered non-NAPs. Scale bar represents 100 μm. **b)** Voltage response from the NAP (red, cell shown in A) and non-NAP (blue) neuron to sinusoidal chirp current injection (black). Below, normalized impedance amplitude profile calculated from voltage responses shown above to the chirp stimulus. Peak resonant frequency is indicated with dashed lines. **c)** Histogram representing distribution of peak resonant frequency for NAP neurons (top, red) and non-NAP neurons (bottom, blue). Resonant frequencies > 2 Hz were designated as ET-like physiology (dashed line), filled bars show counts of ET-like response in both groups). **d)** Proportion of ET-like versus non-ET physiological types in NAP and non-NAP neurons. A chi-square test indicates significant differences in the proportion of neurons having ET-like physiology between groups (χ^2^(1)=16.15, p<0.0001). **e)** Pyramidal neuron morphology of NAPs and non-NAPs revealed using histological processing of neurobiotin fills. Arrows denote neuron recordings from (a & b). Red arrow=NAP; blue arrow= non-NAP. Scale bar represents 100 μm. **f)** Molecular subclass identity of NAP (n=16) nuclei as aligned to Allen Institute taxonomy. **g)** HCN1 transcript counts per million (TPM) in NAPs (n = 13, red) and non-labeled neurons (n = 3, blue) physiologically with L5 ET-like (filled, n = 9) and non-ET like (open, n = 7) physiology. Means are denoted by black bars. Mean HCN1 transcripts counts is significantly higher in ET physiologic type (t=2.49, p=0.026, unpaired t-test, effect size d= 1.22). Statistical significance is denoted by *p<0.05, ****p<0.0001

Although the PFC is broadly connected with other cortical areas,^76^ NAP projections were largely restricted to subcortical regions, with the notable exception of the retrosplenial cortex (Figure 3d, Supplemental Table 1). NAPs innervated subcortical structures known to regulate sleep and anesthesia including the claustrum^77–82^, the nucleus of the diagonal band^83^, and the lateral and preoptic hypothalamus^10,15–17,84^ (Figure 3e–f). NAPs also projected strongly to anterior thalamic nuclei, with minimal labeling to posterior, sensory-related thalamic regions. Within the anterior thalamus, dense labelling was observed in the reticular nucleus, anteromedial, ventromedial and intralaminar nuclei (Figure 4g)—regions critical for the generation of slow waves during sleep and anesthesia.^85–92^

Replication-deficient rabies monosynaptic input tracing^93^ revealed that the majority of NAP inputs were local L5 PFC neurons, with sparse cortical afferents from retrosplenial, motor, and somatosensory areas (Figure 3h–I, Supplemental Table 1). The most prominent subcortical inputs came from anterior thalamic structures, particularly the anteromedial thalamus (Figure 3c–i). Strong inputs were observed from the septal nuclei, nucleus of diagonal band, lateral hypothalamus, and CA1 region of the hippocampus (Figure 3i). These findings support the idea that prefrontal NAPs act as a cortical hub within a distributed brain-wide network regulating sleep and arousal.

### NAPs Exhibit L5 ET-like Physiology

The PFC consists of numerous distinct cell types distinguished by their biophysical properties and synaptic connectivity.^51,50,94^ The specific recruitment of NAP activity during NREM sleep and under anesthesia relative to the nonspecifically labeled L5 PFC neurons raised a possibility that NAPs represent a distinct L5 subtype. Morphological features of NAPs suggested that they are pyramidal neurons (Figure 1n, 4a,e) and their projections are largely subcortical (Figure 3). L5 pyramidal neurons are classified into two broad classes based on whether their projections are contained within the telencephalon (IT) or extend beyond it, referred to as extratelencephalic (ET). IT and ET neurons also possess distinct biophysical properties^94–98^. To determine whether NAPs are enriched in a specific biophysically defined L5 neuronal subtype, we performed targeted whole cell patch clamp recordings in ex vivo slices under quiescent, controlled conditions in the presence of synaptic blockers (Figure 4a).

A key physiological property that distinguishes L5 ET neurons from L5 IT neurons is their somatic subthreshold resonance frequency within the high δ–frequency band (>2 Hz).^95,99^ Consistent with our anatomical data showing that NAPs have extratelecephalic projections, most NAPs were enriched in δ-resonance (23/37) compared to a much smaller proportion of unlabeled L5 pyramidal neurons (3/26) (Figure 4b–e). The somatic resonance profile of ET neurons is caused by hyperpolarization activated cyclic nucleotide gated (HCN1) channels^95,100^. In addition to impedance profiles consistent with greater HCN channel expression (Sup. Figure 8a–f), most NAPs have hallmarks of HCN channels as demonstrated by their response to step current injections: including larger voltage ‘sag’ and rebound responses (Sup. Figure 8g–l). To assess their excitability, we examined the suprathreshold firing properties of NAPs using depolarizing current injections. The initial interspike interval (ISI) was frequently shorter in NAPs compared to neighboring unlabeled neurons, consistent with previous observations of onset doublets in L5 ET neurons in other neocortical brain regions.^101^ In contrast, other suprathreshold properties, including rheobase, voltage threshold for the first spike, maximum rate of rise (dV/dt), and action potential half-width did not significantly differ between NAPs and unlabeled neurons (Sup. Figure 8m–v). Together these data indicate that NAPs are enriched in L5 ET-like physiological properties.

### Single Nucleus RNA Sequencing Confirms that NAPs are Enriched in ET Neuronal Subtype

To further assess the cell-type identity of NAPs, we collected the nuclei in a subset of recordings (16 NAPs and 3 unlabeled neurons) and performed single-cell RNA sequencing to map their transcriptomic subclass. NAP nuclei were mapped using correlation-based clustering to Allen Institute cell type taxonomy. Consistent with the physiological data, most NAP nuclei were molecularly defined as the L5 ET subclass (11/16) (Figure 4f). The remainder of nuclei mapped to the L5–6 IT subclass (5/16). No nuclei mapped to the near-projecting (NP), or L6 CT or L6b molecular subclasses. Given the 35% prevalence of L5 ET transcriptomic subclass in infragranular layers of ventral PFC,^49^ the probability of observing the L5 ET subclass in 11 out of 16 NAPs by chance is 0.0049. Consistent with HCN dependent properties being a key feature of L5 ET physiology, HCN1 gene expression was higher in neurons with L5 ET-physiological properties (Figure 4g). Thus, converging gene expression and neurophysiological data indicate that NAPs are enriched for L5 ET properties. Remarkably, while NAPs are engaged in strong bidirectional interactions with the thalamus, not a single NAP was mapped onto cortico-thalamic (CT) type.

### Prefrontal Colgalt2-Expressing Neurons Recapitulate Prefrontal NAPs Circuit Actions

Based on the convergence of biophysical and gene expression data, we hypothesized that a molecularly defined L5 ET subtype would recapitulate functional and anatomical features of NAPs. To test this, we systematically tested cre-driver lines that label distinct L5 subtypes, including RBP4-cre (L5 IT/ET), TLX3-cre (L5 IT) and Colgalt2-cre (L5 ET),^102^ to determine whether ET-specific subtypes reproduce the distinctive activation preference of NAPs under anesthesia.

**Figure 5:**
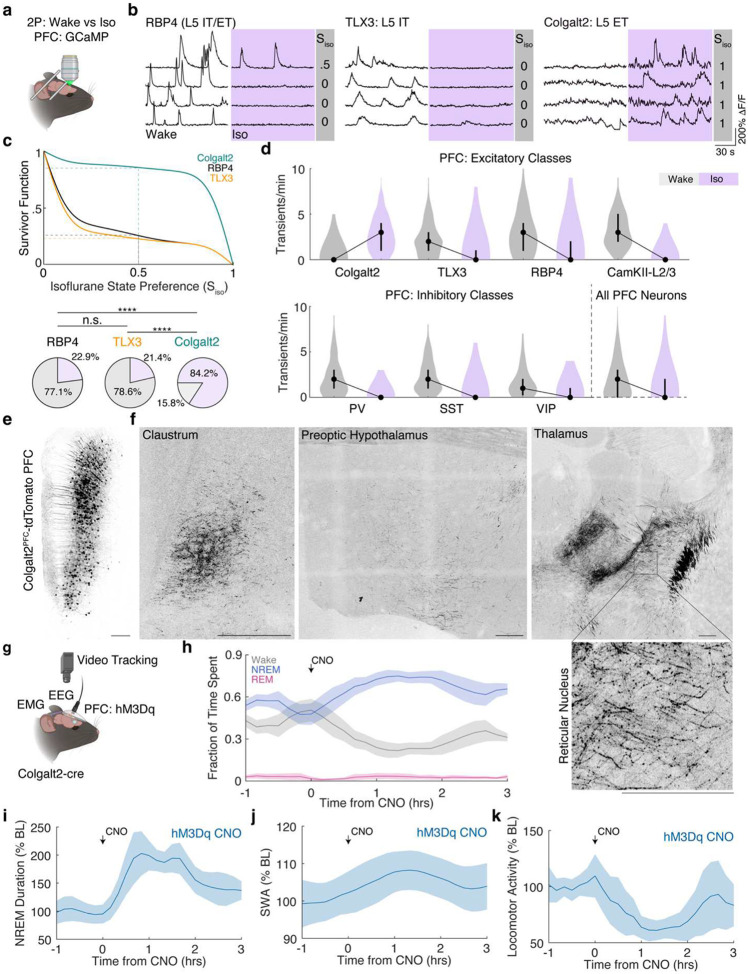
Colgalt2-cre PFC neurons recapitulate prefrontal NAP features. **a)** Schematic of two-photon calcium imaging setup for recording GCaMP signals in prefrontal cortex during wakefulness and isoflurane anesthesia. **b)** Representative calcium activity traces from molecularly defined Layer 5 subtypes shown during wakefulness and under 0.6 % isoflurane (purple): RBP4 (intratelencephalic/ extratelencephalic), TLX3 (intratelencephalic), and Colgalt2 (extratelencephalic), showing divergent state-dependent activity across wake and isoflurane conditions. Numbers shown at right margin indicate Isoflurane State Preference (S_iso_) for each neuron (RBP4 n=4 mice, 170 neurons, TLX3 n=4 mice, 192 neurons, Cogalt2 n=4 mice, 190 neurons). **c)** Survivor function (P) of S_iso_ across the three groups. P(0.5) reflects the fraction of neurons that had higher activity under isoflurane (dashed lines). Binarized pie charts of preferred activity state. A chi-square test indicates significant differences in the proportion of neurons that were preferentially active under isoflurane (χ^2^(2)=195.7, p<0.0001. Post hoc Fisher’s exact tests with Bonferroni correction; RBP4 vs TLX3, p=0.7998; RBP4 vs Colgalt2, p<0.0001; TLX3 vs Colgalt2, p<0.0001). d) Calcium transient rates across prefrontal excitatory (top), inhibitory (bottom), and combined across all subtypes. Note that Colgalt2 is the only molecularly defined subtype that increases calcium activity under anesthesia. Within cell-type statistical comparisons across state performed with sign-rank test (Colgalt2 Wake < Iso, p<0.0001; TLX3 Wake > Iso, p<0.0001; RBP4 Wake > Iso, p<0.0001; CamKII-L2/3 Wake > Iso, p<0.0001; PV Wake > Iso, p<0.0001; SST Wake > Iso, p<0.0001; VIP Wake > Iso, p=0.0039; All PFC Wake > Iso, p<0.0001). Isoflurane-specific median calcium activity significantly varied across groups, with Colgalt2 being significantly more active under isoflurane than all other groups. (H=277.8, df=8, p<0.0001, Kruskal-Wallis test. Dunn’s Multiple Comparisons Test: Colgalt2 vs TLX3, RBP4, CaMKII-L2/3, PV, SST, VIP, All PFC, all p<0.0001). **e)** tdTomato labeleling of Colgalt2 expressing neurons in the PFC. **f)** Representative axonal projection patterns of Colgalt2^PFC^ neurons showing dense terminal fields in claustrum, sparse terminal fields in preoptic hypothalamus, and dense terminal fields in thalamus. Thalamic inset demonstrates fine axon terminals in the reticular thalamic nucleus. Scale bars represent 200 μm in e-f. **g)** Experimental schematic for recording EEG/EMG in Colgalt2 mice expressing-hM3Dq in PFC (n=5). **h)** Fraction of time spent in each of the three states (Wake, NREM, REM). Wake is significantly reduced, NREM is significantly increased, and REM is unchanged following CNO (F(6,48)=53.24, p<0.0001, 2-way ANOVA, Šídák’s multiple comparisons test: Wake BL vs 0–1hr Post CNO, p=0.0004; BL vs 1–2hr Post CNO, p<0.0001; BL vs 2–3hr Post CNO, p<0.0001; NREM BL vs 0–1hr Post CNO, p<0.0001; BL vs 1–2hr Post CNO, p<0.0001; BL vs 2–3hr Post CNO, p<0.0001; REM BL vs 0–1hr Post CNO, p=0.99; BL vs 1–2hr Post CNO, p=0.99 BL vs 2–3hr Post CNO, p=0.99). **i)** hM3Dq reactivation significantly increased NREM sleep duration (F(3,16)=41.39, p<0.0001, 1-way ANOVA; Dunnett’s multiple comparisons test: BL vs 0–1hr Post CNO, p<0.0001; BL vs 1–2hr Post CNO, p<0.0001; BL vs 2–3hr Post CNO, p=0.0001) and **j)** cortical slow-wave activity (F(3,16)=5.564, p=0.0083, 1-way ANOVA; Dunnett’s multiple comparisons test: BL vs 0–1hr Post CNO, p=0.0475; BL vs 1–2hr Post CNO, p=0.0027; BL vs 2–3hr Post CNO, p=0.1034) while significantly decreasing **k)** locomotor activity (F(3,16)=15.57, p<0.0001, 1-way ANOVA; Dunnett’s multiple comparisons test: BL vs 0–1hr Post CNO, p=0.0212; BL vs 1–2hr Post CNO, p<0.0001; BL vs 2–3hr Post CNO, p=0.0134) relative to baseline conditions (mean ± 95% CI shown). Schematics from a, g were created in BioRender.

Spontaneous calcium activity was recorded during wakefulness and under isoflurane anesthesia by expressing GCaMP in the PFC of each line (Figure 5a). Consistent with results in Figure 1, only a small subset of the broad L5, RBP4-expressing neurons exhibited calcium transients under isoflurane. L5 IT TLX3 neurons exhibited a similar activity profile that was less active under isoflurane. In contrast, most L5 ET neurons exhibited strong activity preference under anesthesia (84.2%), compared to RBP4 (22.9%), or TLX3 (21.4%) neurons (Figure 5b, c). In fact, L5-ET cohort was more enriched for anesthetic-preferring neurons than those originally TRAPed under anesthesia (p<0.0001, Fischer’s Exact test). Of all major molecularly defined subtypes of excitatory and inhibitory PFC neurons, L5-ET neurons marked in Colgalt2-cre mice were the only population that increased activity under anesthesia (Figure 5d).

We next examined whether Colgalt2 neurons in the PFC exhibit subcortical projection patterns characteristic of NAPs. Colgalt2-labeled PFC neurons were concentrated in L5 of the PFC (Figure 5e). As with NAPs, Colgalt2 axonal projections were identified in the claustrum^103^. Furthermore, consistent with NAP projections to the thalamus, we identified strong Colgalt2 projections to the anterior thalamic nuclei including the anteromedial and the anterior portion of the reticular nucleus (Figure 5f). Bouton-like structures observed in the reticular nucleus are indicative of potential synaptic sites^104^. While NAPs had a strong projection to the hypothalamus, Colgalt2 projection to the preoptic hypothalamus was comparatively sparse.

To determine whether L5-ET neurons can promote NREM sleep, Colgalt2-cre mice expressing hM3Dq in the PFC were implanted with EEG/EMG (Figure 5 g), and sleep-wake was recorded before and after CNO injection. Chemogenetic activation of L5-ET in the PFC selectively increased NREM sleep over wakefulness, without altering REM sleep (Figure 5h). NREM sleep following L5-ET activation was characterized by longer NREM bout durations, enhanced slow-wave activity, and reduced locomotion (Figure 5 i–k). Together, these results confirm that L5-ET prefrontal neurons recapitulate the unique activity profile under anesthesia and their potentiating effects on NREM sleep of NAPs.

## Discussion

Here we showed that increased activity of L5-ET neurons in the PFC leads to the suppression of arousal. L5-ET neurons are selectively activated in naturally occurring and pharmacologically induced states of diminished consciousness while activity of all other major neuronal populations is suppressed. L5-ET activation is sufficient to promote deep NREM sleep and potentiate effects of anesthesia, while their inhibition partially antagonizes anesthetic-induced slow waves. Unlike most other types of PFC neurons that communicate extensively with other cortical regions, we find that NAPs are primarily synaptically engaged with subcortical structures involved in the regulation of sleep and arousal.

Our findings that PFC plays a key role in suppressing consciousness are consistent with the fact that slow waves during NREM sleep^105^ and under anesthesia^8^ are initiated frontally. These results align with prior work showing that enhancing excitatory synapses in the PFC promotes NREM sleep^40^, while partially silencing L5 synapses^42^ impairs sleep homeostasis, supporting the role of a distinct PFC L5 population in NREM regulation. Yet, other work on the role of the PFC in NREM sleep emphasized the dominant role of the thalamus^89^. We find that NAPs bidirectionally interact with the dorsal thalamic nuclei which play a key role in synchronization of slow waves across the neocortex^89^. Determining whether NAP activity precedes thalamic slow waves and the establishment of the specific causal role of thalamic NAP projection in promoting NREM sleep are topics of interest for further studies. The fact that NAP activation is sufficient to elicit NREM characterized by large amplitude slow oscillations and bidirectionally control their amplitude under anesthesia, however, strongly suggests that the PFC plays a key role in producing slow oscillations that define states of reduced arousal.

Although NAPs show strong bidirectional interactions with the thalamus, our limited transcriptomic analyses revealed no cortico-thalamic (CT) neurons, consistent with predominant localization of NAPs in L5 rather than L6. Interestingly, recent work has begun to elucidate unique features of PFC-thalamic loops that distinguish them from other cortical regions. In the PFC, L5 ET neurons directly excite the reticular nucleus (RT) of the thalamus^89^, a connection observed predominantly for L6 CT neurons elsewhere in the cortex^106^. Consistent with this work, we observe strong NAP projections to the RT (Figure 5). Burst firing of RT neurons is correlated with UP states observed in the LFP under anesthesia and L5 projections to the RT play a key role in this cortico-thalamic synchronization^104^. Large calcium transients observed in NAPs under anesthesia and during NREM sleep may therefore drive RT bursts linked to the generation of slow waves^107,108^. Indirect evidence suggests that L5 cortical neurons synapse on a specific subtype of RT neurons that project to “higher-order” thalamic nuclei rather than primary sensory thalamic nuclei^109^. It is known that physiologically and transcriptomically divergent RT neurons have different effects on NREM sleep^110^. Determining whether NAPs engage a specific RT neuron subtype may reveal the functional implications of the unique PFC-thalamic loop in the control of sleep and arousal.

Recent work has revealed that inhibition of RT neurons by lateral hypothalamic (LH) GABAergic neurons triggers awakening from sleep^111^. Thus, NAPs and wake promoting hypothalamic neurons converge on the RT and exert opposing effects on its activity, suggesting a key role of this circuit in regulation of levels of consciousness, a hypothesis supported by computational models of anesthetic induced unconsciousness^112^. Notably, NAPs are also bidirectionally connected with LH, but the specific identity of LH synaptic partners of NAPs is currently unknown. Finally, NREM-promoting PFC SST neurons inhibit LH GABAergic neurons^37^, and may therefore release RT neurons from inhibition. Together these results outline the specific cell types that comprise a unique PFC-thalamic loop integrated with ascending modulatory hypothalamic nuclei to control the level of consciousness.

NAPs have a strong projection to the claustrum – a structure involved in coordinating synchronous slow wave activity across evolution from reptiles to humans^77–79,82^. Wide projections of the claustrum throughout the neocortex have been used to suggest that claustrum plays a key role in the regulation of conciousness^113^, a controversial claim^114^. While L5-ET projections to the hypothalamus and the brain stem were sparse, it is possible that these also contribute to their arousal suppressing properties. Recent evidence suggests that a single PFC neuron can innervate distant cortical sites^75^ and it is at present unclear whether individual NAPs project to multiple targets or whether distinct targets are contacted by NAP sub-populations. In any event, it appears that NAPs play a central role in broadly distributed network that controls arousal.

Previous work on subcortical circuits mediating effects of anesthesia shows that anesthetics activate sleep-promoting neurons^10,13,14,16,17,67,84,115–118^. Our results here show that the convergence between sleep and anesthesia circuits extends to the neocortex. Remarkably, most known effects of anesthetics on cortical neurons^18,19^ and synapses^119,120^ are inhibitory. Consistent with this widespread inhibition, most PFC neurons, and cortical neurons in general, are suppressed under anesthesia. PFC L5 ET neurons are the only major neuronal subtype that increases activity under anesthesia, pointing to their unique properties. There are several molecular anesthetic mechanisms that could, in principal, enable anesthetics to directly depolarize NAPs^16,121–123^. Identification of NAPs as a specific transcriptomic subtype of L5 neurons, together with the availability of extensive cell-type specific gene expression datasets^49,50^, opens the door for identifying specific mechanisms that lead to NAP activation under anesthesia. Different anesthetics act on distinct molecular targets to reduce consciousness. Whether NAPs serve as a common cellular target for mechanistically diverse anesthetics remains an important question for future research. Understanding the specific molecular mechanisms that lead to NAP activation under anesthesia may have clinical implications for developing novel sedatives and for alleviating PFC dysfunction after anesthesia^124,125^.

Interestingly, previous work in humans and in animal models singled out anesthetic induced suppression of the PFC as critical for disruption of consciousness. Consistent with this view, loss of consciousness induced with variety of anesthetics in humans is associated with changes in frequency and coherence of frontal EEG signals^126,127^ and disruption of fronto-parietal functional connectivity^128–130^. Cholinergic activation of the PFC can precipitate emergence from anesthesia^131^ while inhibition of the PFC delays anesthetic emergence^132^ and decreases wakefulness^133^. Furthermore, cell-type agnostic chemogenetic inhibition of glutamatergic neurons in the PFC potentiated anesthetic effects while excitation reduced anesthetic potency^20^. These findings have been interpreted as lending support to the Global Neuronal Workspace theory of consciousness^134^ and implicate the PFC as a key arousal node^135^. Interestingly, together with previous findings^37^ this work strongly implies that PFC contains both arousal promoting and arousal suppressing microcircuits composed of distinct neuronal subtypes, consistent with the fact that PFC is a highly heterogeneous structure^136^. Understanding the interplay between these microcircuits is essential for deeper insights into mechanisms that control the level of consciousness.

It has been proposed^23^ that the primary effect of anesthetics at the cellular level is the decoupling of dendritic from somatic activity in L5 neurons. Consistent with this hypothesis, both IT and ET L5 neurons exhibit synchronous somatic calcium fluctuations dissociated from dendritic signals^24^. Rather than focusing on relatively small amplitude fast calcium dynamics which may reflect UP and DOWN states^24^, here we focused on large calcium transients thought to be related to nonlinear dendritic integration events^137^ associated with burst firing^138^. Our findings reveal that L5 ET PFC neurons are the only major cortical neuronal subtype to preferentially exhibit large calcium bursts under anesthesia, as corroborated by c-Fos expression, concentrated predominantly in the L5 of the PFC under anesthesia. We did not directly address the relationship between the dendritic and somatic signals in NAPs. Hence, it remains unclear whether uncoupling of dendritic and somatic signals also occurs in the PFC. However it is notable that L5 ET and L5 IT neurons exhibit distinct dendritic physiology that could contribute to the cell-type differences that we observe^97,98^.

All animal behaviors are hierarchically organized into orderly sequence of phases^139,140^. Prior to the onset of physiologically defined sleep, animals exhibit a set of preparatory behaviors^37,141^. Upon their completion, sleep itself proceeds in orderly stages from progressively deepening NREM sleep to REM^88^. It now seems clear that the PFC contains distinct neuronal populations that control all sleep stages: SST neurons that initiate preparatory behaviors^37^, L5 ET neurons that promote NREM sleep, and the hypothalamic projecting PFC glutamatergic neurons that initiate REM^142^. Understanding the dynamics of the PFC microcircuits that interlink these neuronal populations will reveal the mechanics of the orderly progression through different behavioral states that comprise sleep.

There is, however, a deeper level at which sleep needs to be understood. The decision to sleep, the simplest decision made by animals across evolution, must be integrated within the broader behavioral hierarchy. Even relatively “simple” animals must decide whether it is safe to sleep, and this requires executive function that weighs homeostatic and circadian sleep pressures against other behavioral needs. It is therefore unsurprising that the PFC emerges as a central cortical hub that controls sleep. In most studies, including this one, sleep is studied in a highly contrived lab environment where the decision of whether to sleep or to stay awake lacks behavioral consequences. Understanding how the PFC implements the decision to sleep at the expense of all other behaviors will require interrogation of the PFC circuits in more naturalistic settings.

## Materials and Methods

All experiments were approved by the Institutional Animal Care and Use Committee at the University of Pennsylvania (IACUC protocol # 803844, 804902, and 807237). All ex-vivo slice experiments were performed in accordance with Institutional Animal Care and Use Committee at the University of Washington (IACUC protocol # 4586–01). Male and female mice aged 12–46 weeks were equally distributed across all experiments. All experiments were conducted in accordance with the National Institutes of Health guidelines. Mice were housed on a 12h:12h reverse light dark cycle (ZT0/lights on at 9:00pm) with *ad libitum* access to food and water. Table 1 lists all the mouse strains used in this work. Table 2 lists all viruses used in this work.

### General surgical procedures:

All surgeries were performed following IACUC guidelines for rodent survival surgery. Mice were induced with 2.50% isoflurane in 100% oxygen and maintained at 1.50% isoflurane for the duration of the surgery. After a surgical plane of anesthesia was reached, as measured by the lack of a toe pinch response, mice were placed in a stereotactic frame (Kopf Model 940). Core body temperature was regulated with a closed-loop heating pad (CWE Inc, TC1000), and ophthalmic ointment was applied to protect the eyes. All surgeries were performed with aseptic technique and sterile instruments. All animals were administered analgesics and antibiotics along with fluids intraperitoneally prior to surgical incision.

### Stereotaxic Viral Delivery into the PFC:

To target the medial prefrontal cortex, the skull was leveled to accommodate stereotactic coordinates.^143^ Burr holes were created with a stereotaxic microdrill (Stoelting, 51449) using 0.5 mm diameter drill bit (Fine Science Tools, 190070–05). Viruses were delivered using a 32 gauge, 10μL syringe (Hamilton, 80008) mounted on a stereotactic microinjection syringe pump (Stoelting Co, 53311). PFC virus injection coordinates used were: AP 1.70mm, ML 0.30mm, DV −3.00mm relative to bregma. Prior to and following viral injection, the syringe was held in position for 10 minutes to lessen tissue trauma and to avoid virus reflux up the syringe tract.^144^ Following injection, burr holes were filled with bone wax (Fine Science Tools, 19009–00) and the incision was sutured closed. Mice were allowed a minimum of 1 week recovery time prior to any other experimentation.

*Chemogenetic actuator viruses:* Bilateral (300nL per side) injections of either AAV9-Syn-flex-PSAM4-GlyR-IRES-EGFP, AAV9-Syn-flex-PSAM4-GlyR-IRES-EGFP or AAV8-hSyn-DIO-mCherry (excitation, inhibition, and control respectively) were delivered. *Anterograde Projection viruses:* Unilateral 300 nL injection of AAV9-CAG-FLEX-tdTomato was performed. *Monosynaptic input viruses:* 300 nL of AAV9.CMV.FLEX.TVA.mCherry.2A.oG helper virus was injected into the PFC unilaterally. After 2 weeks of viral expression, 1μL EnvA G-deleted Rabies-EGFP was injected at the same stereotaxic coordinates. *Calcium imaging in the PFC:* For GRIN mediated imaging of the PFC, 300 nL of pGP-AAV9-Syn-Flex-JGCaMP8s was injected into the PFC unilaterally.

### EEG/EMG Electrode Implantation

Electrodes were constructed and implanted as previously described.^145^ Briefly, ten epidural EEG leads (Digikey, 853-93-012-10-001000-ND) were implanted 0.65mm lateral to bregma in both hemispheres, ranging from 2.30 mm anterior to bregma to −2.90 mm posterior to bregma. 2 stainless steel EMG wires were soldered onto two separate headpiece connecter leads and were implanted in the dorsal neck muscles (A-M Systems, 793500). Each insulated wire coating was stripped prior to soldering, followed by a reinsulating nail polish coating. Headpieces were secured using dental cement (A-M Systems, 525000 and 526000) and two anchor screws (McMaster-Carr, 91800A050), positioned at 2.50 mm lateral and 2.00 mm posterior to Bregma. Following surgery, mice were singly housed and were allowed to recover from the implant for a minimum of one week prior to investigation. For EEG recordings in animals undergoing chemogenetic modulation, electrode implantation was performed at least one week following the isoTRAP protocol (see below).

### Combined EEG and PFC Calcium Imaging in Awake Behaving Animals:

For simultaneous EEG and one-photon miniscope recordings, we constructed a custom 2×2 headpiece connector by trimming the 2×6 EEG connector to accommodate the miniscope. Modified EEG electrodes were made using stainless steel screws (McMaster-Carr, 91800A052) connected to headpiece via silver wire wrap (A-M Systems, 787000) and solder. Two stainless steel EMG wires were soldered to separate leads of the headpiece connector as above.

Standard V4 variant 2 baseplate (Open-Ephys, OEPS-7416) was modified to enable real-time visualization of GCaMP expressing neurons within the field-of-view (FOV) during GRIN lens implantation. Specifically, we removed the portion of the baseplate cap that normally fits inside of the metal baseplate. Then we drilled a 3.0 mm diameter recessed hole in the center of the modified cap to a depth of 1.2mm, the approximate working distance of the miniscope. A second, precision reamed through-hole with a 0.51mm diameter was drilled, centered about the recessed hole. To ensure precise centering of the GRIN lens within the baseplate, a second baseplate cap with a similar centered, 0.51 mm precision reamed through hole served as a template alignment tool. This template cap was secured to a metal baseplate in its standard orientation and was used to align the modified baseplate cap with a precision 0.51mm pin gauge (McMaster-Carr, 2326A312) inserted through both template cap and modified cap. The recessed circular portion of the modified cap was confirmed to be oriented upwards and then secured to the underside of the metal baseplate with cyanoacrylate adhesive (McMaster-Carr, 7520A12). With the alignment cap and pin gauge removed, a 0.5mm GRIN lens (Inscopix, 1050–004600, 8.4mm length) was press-fit, flush with the top of the recessed area and secured with cyanoacrylate adhesive.

GRIN-lens and EEG/EMG electrode implantation surgery was performed at least one week following the completion of the isoTRAP protocol. Prior to surgical incision, mice were administered 0.2 mg/kg dexamethasone IP. Burr holes were drilled centered at AP −2.0 mm and ± 2.0 mm for EEG screws, AP +3.0 mm, ML 1.7 mm for an additional anchor screw, and AP +2.0 mm, ML −0.3 to −0.4 mm for GRIN lens. The mediolateral coordinate for the GRIN lens was adjusted for each mouse to avoid injury to the sagittal sinus and reduce intraoperative bleeding risk. EEG and anchor screws were secured, followed by EMG wire insertion in the dorsal neck muscles. Next followed stereotactic aspiration of brain tissue above infralimbic area to a depth of ≈1.5mm using a 25G blunt needle, as previously described.^146^ The cavity was perfused with sterile saline and irrigated thoroughly until the effluent became clear. For modified baseplate/GRIN implantation, we utilized the stereotaxic miniscope holder designed by the Aharoni Lab (https://github.com/Aharoni-Lab/Miniscope-v4/tree/master/Miniscope-v4-Holder). The miniscope was mounted onto the modified baseplate with the GRIN lens, allowing real-time visualization of the FOV during implantation. The lens was slowly lowered to a depth of approximately 3.0 mm while monitoring the FOV. The final position was determined when individual cells around this depth (DV −2.8mm to −3.0 mm) became visible. GRIN lens was then secured by layering cyanoacrylate gel adhesive rapidly cured with cyanoacrylate accelerator, cyanoacrylate adhesive, and dental cement, with adequate drying times between. Following surgery, mice were singly housed and were allowed to recover from the implant for a minimum of three weeks to allow for window clearance prior to additional studies. Dexamethasone, cefazolin, and meloxicam were administered for the first 3 post-operative days.

### *In vivo* two photon imaging:

#### Calcium indicator Expression

Two-photon in-vivo calcium imaging studies were performed using AAV9-CAG-Flex-GCaMP6f-WPRE-SV40 in specific neuronal populations labeled in transgenic mouse lines (Table 1). To attain pan-neuronal imaging, pyramidal neurons were labeled using pENN-AAV9-CamKII-GCaMP6f-WPRE-SV40 and AAV9-CAG-Flex-GCaMP6f-WPRE-SV40, or with AAV1-Syn-GCaMP6f-WPRE-SV40. GCaMP6f transfection was achieved using intracranial AAV injections of neonatal mice as previously described.^122^

#### Cranial Window Implantation

1.5–3 months following viral transfection head-mount and acute cranial windows implant surgery was performed as previously described.^147^ Briefly, periosteal tissue across the skull was removed without damaging either temporal or occipital muscles. A mounting bracket consisting of two parallel metal bars was attached to the animal’s skull to aid in head restraint and reduce motion artifacts during imaging. Mice were checked for GCaMP expression, and a round glass coverslip (Thomas Scientific, 1217N66) was implanted and secured with cyanoacrylate adhesive and adhesive resin cement (Parkell, S380). Mice were habituated to head restraint twice daily for 30 minutes on postoperative day 1 and 2 in a custom-built body support to reduce stress.^147^ No obvious distress was observed during the habituation periods across all experimental setups.

#### 2-Photon Imaging

On the day of imaging, awake mice were positioned in the custom head holder device under the two-photon microscope. In vivo two-photon imaging was performed with an Olympus DIY RS two-photon system (tuned to 910–920 nm) equipped with a Coherent Discovery NX laser. Pyramidal neurons in ACC region were recorded for 2-min sessions under awake conditions and once again after 0.6% isoflurane while maintained normothermic with a heating pad. Isoflurane imaging began 30 minutes after anesthetic induction to ensure steady-state conditions. Motion-related artifacts were typically <2 μm. Vertical movements were rare and minimized using two metal bars affixed to the skull (described above) in combination with a custom-built body support.^147^ All experiments were performed using a ×20 Olympus objective (XLUMPLFLN; NA = 1.00, 2.0 mm working distance) immersed in aCSF, with ×2 digital zoom. Images were acquired at a frame rate of 2–4 Hz (2-μs pixel dwell time). Image acquisition was performed using Olympus Fluoview software and analyzed post hoc using ImageJ software version 2.1.0.

### Isoflurane TRAP (isoTRAP) Exposure Protocol to Label Neurons Active Under Anesthesia

This protocol was performed 1–2 weeks following stereotaxic injection of cre-dependent virus into the PFC (see above) to permanently label neurons active under isoflurane anesthesia with a construct of interest. TRAP2-cre mice were habituated to gastight, temperature-controlled, 200mL cylindrical chambers with 100% oxygen flowing at 200 mL/min for two hours per day for three days prior to experimentation, as previously described.^148^ After 1-hour pretreatment with 0.6% isoflurane, mice were injected with 4-hydroxytamoxifen (4-OHT, 40mg/kg IP) to drive nuclear expression of Cre and enable cre-mediated recombination in isoflurane active neurons. Time exposed to open air during IP injections was less than 20 seconds per mouse.^67^ Mice remained exposed to 0.6% isoflurane for another 6 hours following tamoxifen injection. Anesthetic concentrations were confirmed using a Riken FI-21 refractometer (AM Bickford). Mice were allowed a minimum of 1 week recovery to allow for viral expression prior to any further experimentation. A stock concentration of 5mg/mL of 4-hydroxytamoxifen (Hello Bio, HB6040) solution was prepared by dissolving the 10mg of drug in 0.5 mL 100% ethanol, heating the suspension at 60 C for 10 minutes, then adding 0.5 mL kolliphor oil (Sigma-Adrich, C5135), and lastly adding 1.0 mL 0.1X PBS with 10 minute vortexing period between each step.^149^

### Chemogenetic Agonist Preparation and Dosing

Clozapine-N-oxide, CNO (Sigma-Aldrich, C0832), was dissolved in sterile saline to a final concentration of 0.3 mg/mL. CNO dosing for all experiments was 3.0 mg/kg IP.^150^ uPSEM 817 tartrate (Tocris, 6866) was dissolved in sterile saline to a final concentration of 0.1 mg/mL. uPSEM dosing for all experiments was 1.0 mg/kg.^151^ All drug preparations were prepared fresh for each experiment and all drugs were administered intraperitoneally (IP).

### Repeated Righting Reflex Assessment

Anesthetic was delivered and monitored using the same procedure as above. Righting reflex (RR) assessments were performed under steady state isoflurane 0.6 % conditions^152,153^ as described previously. RR was then assessed every 3 minutes. Successful righting probability was estimated as fraction of all trials on which righting was achieved in sliding windows of 10 trials (window step 1 trial). 95% confidence bounds were estimated using the binomial distribution.

For each chemogenetic manipulation experiment mice first underwent a control injection of saline. Following the saline injection, with isoflurane maintained at 0.60%, the righting reflex was assessed every 3 minutes for 1 hour.^67,152–154^ Mice received an IP CNO injection at the conclusion of the final saline righting reflex assessment. After a 15-minute re-equilibration period, mice were subjected to an additional 3 hours of righting reflex assessments at 3-minute intervals.

### EEG/EMG and Miniscope Recordings

#### EEG recording apparatus

A 32-channel headstage (Intan Technologies, C3324) linked to an acquisition system (Intan Technologies, C3004, controlled with RHX software) was used for all neurophysiological recordings. EEG, EMG and 3-axis headstage accelerometer signals were acquired continuously at 1KHz per channel. The headstage interfaced with the EEG and EMG electrodes via a custom-designed PCB adaptor. All filtering and processing of the EEG signals was performed *post-hoc*. A slip-ring commutator (Digikey, 152–1175-ND) attached to a spring counter-balanced lever arm (Instech, CMMI/65) was used to connect the EEG headstage to the acquisition system using SPI cable adapter boards (Intan Technologies, C3430).

#### GRIN-mediated in vivo Ca imaging apparatus

Miniscope (Open Ephys, v4.4, OEPS-7407) was used to acquire calcium signals sampled at 20 Hz. 5 minutes of continuous recordings were interspersed with 5 minutes of rest to avoid excessive photobleaching and heat damage. LED power was maintained at a constant level. Minimal sufficient LED power was used in all recordings (<1mW). For combined EEG/Miniscope recordings the signals were connected to a motorized commutator (LabMaker, Open-MAC Commutator) enabling unrestricted movement during recordings.^155^ Calcium signals were recorded with a Miniscope-DAQ (Open Ephys, v3.3, OEPS-9050) controlled with Miniscope DAQ QT Software v1.11 (Aharoni Lab, https://github.com/Aharoni-Lab/Miniscope-DAQ-QT-Software/releases).

#### Video tracking of behavior

An infrared LED (Adafruit, 387) was soldered to the EEG headstage and tracked using a USB infrared camera (SVPRO OV2710), modified to operate exclusively under infrared conditions, allowing continuous monitoring of mouse movements. Video camera frames were acquired at 5 Hz. Real-time LED centroid tracking was used to estimate ambulatory activity as described previously.^156^

#### Signal Synchronization

Miniscope images, video camera frames, anesthetic gas concentration, and light: dark cycle, were synchronized to the electrophysiological signals via an I/O expansion module (Intan Technologies, E6500). Synchronization was achieved using TTL pulses delivered by an Arduino Uno (Mouser Electronics, A000066) and managed via custom built MATLAB (MathWorks, 2024a) interface.

#### Anesthesia EEG Experiments

EEG recordings under anesthesia were performed in a gas-tight, temperature controlled, cylindrical recording chamber as described previously in tether-habituated mice.^157^ Briefly, fresh gas was delivered at 8 L/min (approximately one full volume exchange per minute). Body temperature was maintained at 37°C via partial submersion of the chamber in a circulating 37°C water bath. Anesthetic gas concentrations were sampled using a Poet IQ 602–6A Gas Monitor (Criticare Systems). For chemogenetic modulation experiments mice expressing either excitatory (hM3Dq) or inhibitory (PSAM) receptors in the PFC were exposed to 0.4% isoflurane for 1 hour. Then, hM3Dq-expressing and PSAM-expressing mice received CNO or uPSEM respectively (CNO and uPSEM preparation and dosing in: Chemogenetic Agonist Preparation and Dosing section). EEG recordings continued for another 3 hours following chemogenetic agonist delivery. For combined EEG/Calcium imaging experiments, 30 minutes of baseline recordings were followed by 1 hour of 0.6% isoflurane.

#### Sleep/Wake Recordings

All sleep/wake EEG recordings were conducted in a dedicated room in custom-built sound-attenuated recording chambers that fit each animal’s home cage. A 12:12, light-dark cycle synchronized to that used in the animal facility was maintained in each recording chamber. Animals were given access to food and water ad-libitum. Prior to each recordings mice were habituated to the apparatus and tethering for at least 24 hours. This habituation period was followed by 1–24 hrs of baseline recordings. Chemogenetic activation of neurons of interest was performed at ZT=18 (middle of the dark phase) and followed by at least 4 hours of continuous recordings (CNO dosing in: Chemogenetic Agonist Preparation and Dosing section. For sleep/wake EEG experiments with simultaneous miniscope recordings, mice expressing GCaMP were recorded in the same apparatus for a period between 4 and 24 hours.

### Analysis of Electrophysiological Signals

All analysis of electrophysiological signals was performed *post-hoc* using custom Matlab code. EEG signals were decimated to 125 Hz, high pass filtered (4^th^ order Butterworth filter, cutoff frequency of 0.5Hz) and mean re-referenced. EMG signals were high pass filtered (4^th^ order Butterworth filter, cutoff frequency of 300Hz) and differentially referenced. All filtering was performed in both directions to minimize phase delays (MATLAB *filtfilt* function). Multitaper power spectral estimates (35 tapers, 5 second window, 2.5 second step) were computed using Chronux.^158^ EMG and accelerometer RMS was computed in the same temporal windows.

To assess changes in spectral power in anesthesia EEG experiments before and after chemogenetic modulation, we computed the ratio of spectral power at each frequency. 30 minutes of artifact free EEG was used to estimate power spectrum for each mouse for the 30 minutes prior to chemogenetic agonist injection or 60–90 minutes following injection. 95% confidence intervals for resulting power ratios were estimated using jackknife resampling across mice.

To assess changes in sleep/wake, we developed a feedforward, fully connected neural network classification model, using MATLAB’s *fitcnet* function. Input features were extracted from 5-second windows of EEG, EMG, and accelerometer data. Dimensionality of the EEG spectrogram was reduced using non-negative matrix factorization^159^, to six features (91.4% of spectral variance captured). These were augmented with EMG RMS and accelerometer RMS to form an eight-dimensional input vector. The network was trained in a supervised manner using manually scored labels (Wake, NREM, REM) from a subset of data recorded from 16 mice. Training set contained approximately equal proportions of all states. Network classification was validated using a leave-one-mouse-out cross-validation strategy. The neural network classifier achieved an overall training accuracy of 95% and overall testing accuracy (on left out data) of 93% across all folds. (See Sup. Figure 3)

Total time spent in each state, NREM bout duration, and slow wave activity (SWA) were calculated and compared for each state and compared between groups. SWA was defined as the faction of power within the 0.5–4 Hz frequency range. NREM bout duration and SWA were normalized for each animal’s baseline period (1 hour prior to chemogenetic modulation). 95% confidence intervals for resulting comparisons were estimated using jackknife resampling across mice. 1-way or 2-way ANOVAs were used to compare Fraction of Time Spent in each vigilance state, NREM bout durations, SWA, and locomotor activity across 1 hour epochs before and after injection. Lastly, transition probability matrices were estimated during the CNO period (0.5–2.5 hours post-injection) and compared to those estimated during a zeitgeber-matched baseline period 24 hours earlier. 7 hM3Dq and 8 mCherry mice were used for this analysis; excluded mice did not have baseline ZT matched recordings. These transitions matrices were compared with 2-way ANOVAs as well. To eliminate transient fluctuations, classified network states were required to persist for at least fifteen consecutive windows.^160^

### Analysis of Calcium Imaging Recordings

Miniscope videos were spatially decimated by a factor of 2 and temporally downsampled to a frame rate of 4 fps. Video frames were then Gaussian filtered and motion corrected using piecewise rigid alignment (NoRMCorre).^161^ Motion correction results were verified manually, and any motion related artifacts were typically less than 2 μm. Regions of interest (ROIs) corresponding to cell soma were manually selected. For each ROI, average pixel intensities were computed to obtain a single raw-fluorescence time-series. Raw calcium traces were detrended (3^rd^ order polynomial) to remove slow signal drifts and then normalized to their mean, resulting in ΔF/F values for cross cell comparison. Calcium transients were detected using the *findpeaks* function in Matlab. Peaks were required to exceed 100% ΔF/F, have a minimum prominence of 80% ΔF/F, and be separated by at least 1 second.

Two-photon calcium imaging videos were analyzed post hoc using ImageJ software version 2.1.0. All time-lapse images from each individual field of view were motion-corrected and referenced to a single template frame using cross-correlation image alignment (TurboReg plugin for ImageJ version 2.1.0). ROIs corresponding to visually identifiable somas were manually selected from the field of view. For each ROI, pixel intensities were averaged to generate a fluorescence trace. Background fluorescence was estimated as the average pixel value per frame from a region without GCaMP expression (e.g., blood vessel) and subtracted from the ROI trace. Baseline fluorescence (F_0_) was calculated as the mean of inactive periods of the trace (~2 s). Normalized ΔF/F_0_ traces were then used for calcium transient detection, defined as periods where the trace exceeded 3 standard deviations above the mean signal.

#### State Preference Estimation:

Neuronal activity was estimated as the number of calcium transients (see above) per minute of recording. To quantify activity preference for a specific state *i* (e.g. Wake/Isoflurane or Wake, NREM, REM) we used a state preference index $\left(S\right):S_{i}=\frac{F_{i}}{\sum_{j=1}^{n}F_{j}}$ where *F*_*i*_ is the calcium transient frequency (transients/minute) observed in state of interest *(i)*, and the denominator is the sum of frequencies in all states n states in which this neuron was recorded. Thus, a value of 0 indicates a cell with no transients in state *i*, whereas a value of 1 indicates a cell with all transients exclusively in state *i*. *S*_*i*_ was computed for each neuron. Survivor and simplex plots of *S*_*i*_ across all neurons in a specific population are shown in Figures 1, 2, 5, Sup Fig 2 to illustrate the distribution of state preferences in a given neuronal population of interest. The preferred state for each neuron was assigned as the state in which most transients were detected. Neurons in which no transients were observed in any state were excluded from the analysis.

#### c-Fos Localization to Layer 5 Confirmation Studies:

RBP4 x Ai6 mice were exposed to 0.9% isoflurane or oxygen for 2 hours prior to brain harvest. Upon transcardial perfusion, brain harvest and overnight post fix in 4% paraformaldehyde, brains were placed in PBS with 0.02% sodium azide until ready for sectioning. A compresstome (Precisionary Instruments VF-510–0Z) was used to prepare 40μm thick sections for c-Fos and NeuN staining (1:4 series cut, 1 well stained). Sections were blocked in 2.5% normal goat serum in PBST (0.3% Triton X-100 in PBS) for 2 hours at room temperature, followed by overnight incubation at 4C in rabbit anti-c-Fos primary antibody (Cell Signaling Technologies 2250, 1:5000) and mouse anti-NeuN (abcam ab104224, 1:1000) in 2.5% normal goat serum in PBST. Sections were then washed in PBS, followed by a 2-hour incubation with Alexa Fluor 594 goat anti-rabbit (ThermoFisher 1:200) for c-Fos visualization and Alexa Fluor 647 goat anti-mouse (ThermoFisher 1:200) for NeuN visualization. Following a final wash in PBS, sections were mounted and coverslipped with mounting medium containing a DAPI counterstain (Southern Biotech 1011–20). Sections were imaged with a 20x objective lens using a confocal microscope (SP5II, Lecia Microsystems).

NeuN staining was used for automated neuronal soma detection as follows. Grayscale NeuN images underwent neural network classification using Ilastik^162^ using the NucleiSegmentationBoundaryModel from BioImage Model Zoo.^163^ Resultant probability maps were binarized in MATLAB at a threshold probability of 0.9. Region properties of the binarized cell detection map were computed and selected for further analysis using the following thresholds parameters: area threshold (in pixels) 30–1500, circularity .3–1.7, equivalent diameter 5–40. A pixel index list was created for each resulting segmented soma for each image separately.

To automatically determine whether a cell body was positive for c-Fos and/or Ai6, c-Fos and Ai6 channels were independently filtered by a 2-D gaussian smoothing kernel with a standard deviation of 2 pixels. Within identified neuronal cell bodies, presence or absence of stain was classified by a 2-state Gaussian mixture model based on median stain intensity within each NeuN-identified cell body. Posterior probabilities of greater than 0.9 were required to classify a cell as positive. Double labeled cells were identified as the union of c-Fos and ZsGreen positive. Manual regions of interest were drawn by a blinded experimenter to limit analysis to the prelimbic, infralimbic, and cingulate cortex. The number of positive neurons in each region was averaged across laterality.

### Verification of Chemogenetic Modulation of c-Fos Activity

Two hours after application of chemogenetic actuator (CNO or uPSEM), mice were euthanized and brains were perfused and harvested (as above). For c-Fos immunohistochemistry, sections were blocked in 2.5% normal goat serum in PBST (0.3% Triton X-100 in PBS) and incubated overnight at 4 °C with rabbit anti-c-Fos (Cell Signaling Technologies 2250, 1:5000). After PBS washes, sections were incubated with Alexa Fluor 647 goat anti-rabbit secondary antibody (ThermoFisher, 1:200), counterstained with DAPI, and coverslipped. Images were acquired using a Leica SP5II confocal microscope with a 20× objective.

Infralimbic subregions (~1mm^2^) of grayscale c-Fos and viral expression images were processed separately using Ilastik, where Pixel Classification + Object Classification models were trained.^162^ The resulting object identity maps were exported and analyzed in Matlab. Co-localization was quantified by assessing the overlap of c-Fos identified ROIs with somas identified with viral expression.

### Thermal Imaging

Body temperature monitoring was performed in freely moving mice using a thermal imaging camera (FLIR Lepton 3.5, 160×120 pixels) positioned above home cages. Thermal images were acquired via the Lepton PureThermal SDK (v1.0.2) in MATLAB at 5 Hz. Cages contained standard bedding, nesting material, food pellets, and water. Mice were maintained in constant darkness to preserve free-running diurnal cycles. After 24 hr habituation, mice received IP CNO with ≥48 hr of subsequent acquisition.

Frames were down sampled to 0.1 Hz. The mouse was identified as the hottest 1% of pixels per frame, which was verified manually to capture the head and body while excluding cage surfaces. The median temperature of these pixels was computed and smoothed using a 5-min moving median to remove transient spikes caused by flat-field correction. Temperatures were normalized to a reference baseline defined as the mean over a 1 h period ~25 h post-injection. Two time courses were analyzed: post-injection (from CNO administration) and a control period 24 h later at the same ZT to account for diurnal variation. Temperature changes between control and post-CNO periods were compared across hM3Dq and mCherry groups (50 minute windowed centered 90 min post-injection).

### Whole Brain Mapping of Neural Activity, Projections, and Monosynaptic Inputs

#### Viral Expression Timeline

To ensure sufficient expression of the anterograde viral label (tdTomato, see above) animals were euthanized two weeks following isoTRAP protocol. For monosynaptic input tracing, a separate cohort of mice TRAP2-cre mice were first injected with a cre-dependent helper virus into the prefrontal cortex one week prior to isoTRAP exposure. Three weeks following isoTRAP, a genetically modified, replication-deficient rabies virus was injected into the same site to label direct presynaptic inputs of isoflurane active neurons (see above). To ensure optimal expression of neurons infected with G-deleted Rabies-EGFP, the survival time was set to five days following rabies virus injection.^93^ Following viral expression timelines for both projection and input groups, mice were exposed to 0.6% isoflurane for 4 hours. This was done to independently label anesthetic active neurons via c-Fos immunoreactivity. Following this isoflurane exposure, animals were rapidly euthanized and transcardially perfused using ice cold PBS with heparin (10 U/mL) followed by 4% paraformaldehyde. Brains were removed and post-fixed in 4% paraformaldehyde at 4°C overnight. Whole brains were then washed in PBS and shipped to LifeCanvas Technologies (Cambridge, MA) in a PBS with 0.02% sodium azide for further processing, staining, imaging, and mapping.

The c-Fos staining dataset in this study includes 18 brains stained for c-Fos in this study and an additional 10 brains subjected to identical protocol from our prior study from Wasilczuk et al.^67^ (WTM 1–5 and WTF 1–5 datasets found at https://zenodo.org/record/8384835 were incorporated in this analysis). Therefore, a total of 28 brains were used for c-Fos analyses in this paper.

#### Tissue Preservation, Clearing, Immunolabeling, and Imaging

Paraformaldehyde-fixed samples were preserved with SHIELD reagents (LifeCanvas Technologies: LCT) using the manufacturer’s instructions.^58^ Samples were delipidated using LCT Clear+ delipidation reagents. Following delipidation, samples were blocked using Antibody Blocking Solution (LCT) with 5% normal donkey serum (Jackson ImmunoResearch Laboratories Inc.) Samples were then labeled using the c-Fos optimized protocol (LCT) for primary labeling. This was followed by secondary labeling using SmartBatch+ Secondary Buffer system (LCT) with eFLASH^164^ technology which integrates stochastic electrotransport,^59^ using a SmartBatch+ device (LCT). After immunolabeling, samples were incubated in 50% EasyIndex (RI = 1.52, LCT) overnight at 37°C followed by 1 d incubation in 100% EasyIndex for refractive index matching. After index matching the samples were imaged using a SmartSPIM axially-swept light sheet microscope using a 3.6x objective (0.2 NA) (LCT).

#### Atlas Registration and Cell Detection

Samples were registered to the Allen Brain Atlas (Allen Institute: https://portal.brain-map.org/) using an automated process (alignment performed by LCT) at 25 μm × 25 μm × 25 μm resolution (1 voxel: 625μm^3^). For each brain, a chosen channel—NeuN, YoPro (nuclear dye), or autofluorescence—was registered to an average atlas of the same channel (generated by LCT from previously registered samples). Registration was performed using successive rigid, affine, and b-spline warping algorithms (SimpleElastix: https://simpleelastix.github.io/).

Automated cell detection was performed using a custom convolutional neural network through the Tensorflow python package. The cell detection was performed by two networks in sequence. First, a fully-convolutional detection network^61^ based on a U-Net architecture^61^ was used to find possible positive locations. Second, a convolutional network using a ResNet architecture^62^ was used to classify each location as positive or negative. Candidate cell centers were evaluated using watershed-based segmentation to isolate individual structures. Segmented objects were classified as cells if their total volume (including soma and proximal processes) was less than 2000 μm^3^—a threshold empirically determined from true positive examples. Using the previously calculated Atlas Registration, each cell location was projected onto the Allen Brain Atlas in order to count the number of cells for each atlas-defined region.

#### Brain Wide c-Fos Density, Anterograde Projection, and Monosynaptic Input Estimation

c-Fos–positive neurons were assigned voxel coordinates, and density estimates (Figure 1) represent the mean number of neurons per voxel. Cortical region densities were computed by summing voxels corresponding to previously defined “Summary Structures”^63,67^ and layer-specific prefrontal densities were calculated using relevant Allen atlas structures^63^; differences were assessed with repeated measures one-way ANOVAs. Brains expressing tdTomato were similarly registered, and projection intensity estimates (Figure 3) represent mean voxel intensity. To normalize for differences in virus expression and imaging, intensity in each voxel was normalized by the total stain intensity computed over the entire brain excluding the injection site (prefrontal cortex: infralimbic, prelimbic, anterior cingulate) as well as olfactory-associated areas, cerebellum, and orbital regions. Monosynaptic input densities (Figure 3) represent mean input cell counts per voxel, calculated as the fraction of total inputs across the brain excluding the same regions. For connectivity graphs, only structures in the 90th percentile or higher of projection or input fraction were included.

### Ex Vivo Tissue Preparation

Ex vivo recordings from frontal cortex were obtained using 30–46 week old male and female activity-tagged mice (see above) that were deeply anesthetized by IP administration of ketamine (130 mg/kg) and xylazine (8.8 mg/kg) mix and were perfused through the heart with chilled (2–4°C) sodium-free aCSF consisting of (in mM): 210 Sucrose, 7 d-glucose, 25 NaHCO_3,_ 2.5 KCl, 1.25 NaH_2_PO_4_, 7 MgCl_2_, 0.5 CaCl_2,_1.3 Na-ascorbate, 3 Na-pyruvate bubbled with carbogen (95% O_2_/5% CO_2_). Off-coronal slices (15 degrees tilted rostrally) 300 microns thick were generated using a Leica vibratome (VT1200, Leica, Germany) in the same sodium-free aCSF and were transferred to warmed (35°C) holding solution (in mM): 125 NaCl, 2.5 KCl, 1.25 NaH_2_PO_4_, 26 NaHCO_3_, 2 CaCl_2_, 2 MgCl2, 17 dextrose, and 1.3 sodium pyruvate bubbled with carbogen (95% O_2_/5% CO_2_). After 30 minutes of recovery, the chamber holding slices was allowed to cool to room temperature. Slices were maintained under these conditions until transferred to the recording chamber for patch-seq experiments.

#### Patch-Seq Recordings

Prior to data collection all surfaces and equipment were cleaned with RNAse Zap (Sigma-Aldrich) and nuclease-free water. Brain slices were placed in a submerged, heated (32–34°C) chamber that was continuously perfused with fresh, carbogenated aCSF consisting of (in mM): 125 NaCl, 3.0 KCl, 1.25 NaH_2_PO_4_, 26 NaHCO_3_, 2 CaCl_2_, 1 MgCl_2_, 17 dextrose, and 1.3 sodium pyruvate bubbled with carbogen (95% O_2_/5% CO_2_) at 32–35°C. Ionotropic synaptic receptor blockers were included 25 μM d-APV, 20 μM DNQX and 100 μM picrotoxin in all recording solutions. Fluorescently labeled and adjacent neurons were visualized with upright microscopes (Olympus BX51WI/Zeiss Examiner) equipped with infrared differential interference contrast (IR-DIC) or Dodt optics using 40x water immersion objectives. The somatic depth (150 to 700 μm from pia) of targeted neurons depended upon the cytoarchitecture of the cortical layers, which are more compact in ventral prefrontal cortex. Reflected fluorescent light was separated using two dichroic mirrors and through green (ET525/70 m-2P; Chroma) and red (ET650/75 m-2P; Chroma) emission filters. Whole-cell patch recordings were performed with pulled borosilicate glass pipettes (P1000, Sutter, Novato, CA) with tip openings about 1 μm and resistances of 3–7 MΩ, backfilled filled using 5–20 μl of internal recording solution consisting of (in mM): 110.0 K-gluconate, 10.0 HEPES, 0.2 EGTA, 4 KCl, 0.3 Na_2_-GTP, 10 phosphocreatine disodium salt hydrate, 1 Mg-ATP, 20 mg/mL glycogen, 0.5U/mL RNase inhibitor (Takara, 2313A), 0.5% biocytin and 0.02 Alexa 594 or 488 – pH adjusted to 7.3 with KOH. Whole cell somatic recordings were acquired using either an Axoclamp 2B or a Multiclamp 700B amplifier and custom acquisition software written in Igor Pro (MIES or using custom protocols written by Rick Gray and modified by N. Dembrow). Electrical signals were digitized at 20 or 50 kHz by an ITC-18 (HEKA) and were filtered at 10 kHz. The pipette capacitance was compensated, and the bridge was balanced (5–30 MΩ) during the entirety of the current clamp recording. Reported voltages were not corrected for the measured liquid junction potential (8 mV) and recordings with >30 MΩ series resistances or <0 mV spike overshoots were discarded. At the end of the recording, negative pressure was applied to collect the nucleus and was extruded into 10.4 μL collection buffer consisting of SMART-seq Takara lysis buffer, RNase Inhibitor (0.17 U/μl), and ERCC RNA Spike-In Mix 1 (1×10^−8^).

### Processing of Patch-Seq Samples

Patch-seq samples were processed by standardized procedures developed by Allen Institute, as described in Lee et al. 2021^165^ and Dembrow et al. 2024.^96^ SMART-Seq v4 Ultra Low Input RNA Kit for Sequencing (Takara, 634894) and library construction using Nextera XT DNA Library Preparation Kit (Illumina FC-131–1096) were performed by Genewhiz/Azenta, Inc., following Takara kit instructions except at 0.2x reaction size. Samples were sequenced at a minimum of 17 million reads per sample using Illumina MiniSeq with 2×150 bp ends. Sequence reads were trimmed to remove possible adapter sequences and nucleotides with poor quality using Trimmomatic v.0.36. The trimmed reads were mapped to the Mus musculus GRCm38 reference genome available on ENSEMBL using the STAR aligner v.2.5.2b. The STAR aligner is a splice aligner that detects splice junctions and incorporates them to help align the entire read sequences. Unique gene hit counts were calculated by using the Subread package v.1.5.2. Transcripts per kilobase million (TPM) for each gene x sample were then mapped to the mouse 10x Whole mouse brain cell type taxonomy (CCN20230722) using both correlation and hierarchical clustering with MapMyCells (RRID:SCR_024672). Samples were included if they passed all of the following quality control metrics: >10 million mapped unique reads, >6000 genes detected, bootstrap probability at the class level of 1, and >0.45 supertype correlation score.

### Electrophysiological Analyses

Three sets of stimuli were utilized to probe the electrophysiological properties of neurons. First, subthreshold chirp stimuli were sinusoidal current injections that increased logarithmically in frequency from 0.2 to 40 Hz over 20s. The amplitude of the chirp was adjusted for each neuron to produce a peak-to-peak deflection of 5–10 mV. Impedance amplitude profiles (ZAP) were calculated as the ratio of the Fourier transform of the voltage response to the Fourier transform of the chirp. The frequency at which the maximum impedance occurred was defined as the resonant frequency (fR). Resonance strength was defined as the ratio of the maximum impedance to the impedance at .5 Hz. The 3 dB cutoff was defined as the frequency at which the ZAP attenuated to the square root of 0.5 of the maximum impedance. The second set of stimuli comprised 11 subthreshold 1 s square wave current injections from −150 pA to 50 pA in 20 pA steps. Maximum and steady-state input resistance were calculated from the linear portion of the current voltage relationship in response to this protocol. Voltage sag was defined as the ratio of maximum to steady state input resistance. Rebound slope was defined as the slope of the rebound amplitude as a function of steady-state membrane potential. The third stimulus set consisted of a series of 1 s square wave depolarizing current injections that increased in amplitude by +50 pA/step to a maximum of 0.95 nA or until depolarization induced spike failure occurred. Threshold current was defined as the minimum current injection within this series to drive an action potential. Single action potential properties were measured from the voltage response of the first spike from at this current. This included action potential voltage threshold, defined as the voltage where the first derivative of the voltage response exceeded 20 V/s, maximum dV/dt, minimum dV/dt, and action potential width measured at half the amplitude between this threshold and the peak voltage. The first interspike interval (ISI) was measured at the minimal current step that drove at least two action potentials. Spike frequency accommodation (SFA), defined as the ratio of the second ISI to last ISI, was measured from the first spike train that produced at least 10 spikes.

### Statistical Analyses

Animals were randomly assigned to experimental groups. Data were processed and analyzed in Matlab (2024a, Mathworks, Natick MA) using the Signal Processing, Image Processing, Curve Fitting, Deep Learning, Parallel Processing, and Statistics and Machine Learning Toolboxes. EEG spectral estimation was performed using Chronux open source software.^166^ Statistical comparisons were performed using Prism 10.6.0 (GraphPad Software, San Diego, CA, USA). Data were tested for normality, and appropriate parametric or nonparametric statistical comparison tests were used and reported within the figure legends. Reported values are (mean ± 95% CI) unless noted otherwise.

For all ex-vivo electrophysiology group comparisons, statistical testing was done using Igor Pro. Normality was tested using a Kolmogorov-Smirnov (KS) test. If the D statistic was larger than the critical value, normality was not assumed, and a Wilcoxon Rank test was used for comparison. D statistics smaller than the critical value were assessed as normally distributed, then tested for equal variances in the two samples, and then compared with a two-sample t-test. For all groups, a Cohen’s d statistic was also performed to measure the effect size of the difference between groups. P < 0.05 was considered statistically significant for all comparisons with appropriate corrections for multiple comparisons.

## Supplementary Material

1

## Figures and Tables

**Table 1:** Mouse strains used in this work.

Mouse Line	Source	Description/Purpose
C57BL/6J	JAX: 000664	Wild type
TRAP2-cre (*Fos*^*tm2.1(icre/ERT2)Luo*^/J)	JAX: 030323	Activity-tagging neurons
PV-cre (B6;129P2-*Pvalb*^*tm1(cre)Arbr*^/J)	JAX: 008069	Interneuron line
SST-cre (*Sst*^*tm2.1(cre)Zjh*^/J)	JAX: 013044	Interneuron line
VIP-cre (*Vip*^*tm1(cre)Zjh*^/J)	JAX: 010908	Interneuron line
Ai6 (B6.Cg-*Gt(ROSA)26Sor*^*tm6(CAG-ZsGreen1)Hze*^/J)	JAX: 007906	Fluorescent reporter
RBP4-cre (Tg(Rbp4-cre)KL100Gsat/Mmcd)	MMRC: 031125	Layer 5 line
TLX3-cre (B6.FVB(Cg)-Tg(Tlx3-cre)PL56Gsat/Mmucd)	MMRC: 041158	Layer 5 IT-neuron line
Colgalt2-cre (Tg(Glt25d2-cre)NF107Gsat/Mmucd)	MMRC: 036504	Layer 5 ET-neuron line

**Table 2:** Viruses used in this work.

Virus Name	Source	Concentration (GC/ml)	Description/Purpose
*Chemogenetics*			
AAV8-hSyn-DIO-hM3Dq-mCherry	Addgene: 44361	2.1 × 10^13^	Chemogenetic excitation
AAV9-Syn-flex-PSAM4-GlyR-IRES-EGFP	Addgene: 119741	2.1 × 10^13^	Chemogenetic inhibition
AAV8-hSyn-DIO-mCherry	Addgene: 50459	2.2 × 10^13^	Chemogenetic control
*Anatomic Tracing*			
AAV9-CAG-FLEX-tdTomato	Addgene: 28306	3.1 × 10^13^	Anterograde tracing
AAV9.CMV.FLEX.TVA.mCherry.2A.oG	Salk: 102985	1.37 × 10^13^	Monosynaptic input tracing: helper adenovirus
EnvA G-deleted Rabies-EGFP	Salk: 32635	6.1 × 10^8^	Monosynaptic input tracing: modified rabies virus
*Calcium Imaging*			
pGP-AAV9-Syn-Flex-JGCaMP8s	Addgene: 162377	2.3 × 10^13^	1P miniscope imaging
AAV9-CAG-Flex-GCaMP6f-WPRE-SV40	Addgene: 100835	2.1 × 10^13^	2P imaging, specific neuronal populations
AAV1-hSyn-GCaMP6f-WPRE-SV40	Addgene: 100837	2.2 × 10^13^	2P imaging: pan-neuronal
pENN-AAV9-CamKII-Cre-WPRE-SV40	Addgene: 105551	1.1 × 10^13^	2P imaging: pyramidal neurons
