# Supplementary material for: Convergent Control of NREM Sleep and Anesthesia by Prefrontal Layer 5 Extratelencephalic Neurons
Source: Res Sq. 2025 Nov 19:rs.3.rs-7861434. Preprint. [Version 1] doi: 10.21203/rs.3.rs-7861434/v1 (PMC12668140; doi:10.21203/rs.3.rs-7861434/v1)
Supplement: 1 [file NIHPPRS7861434V1-supplement-1.pdf]

Supplemental Figures

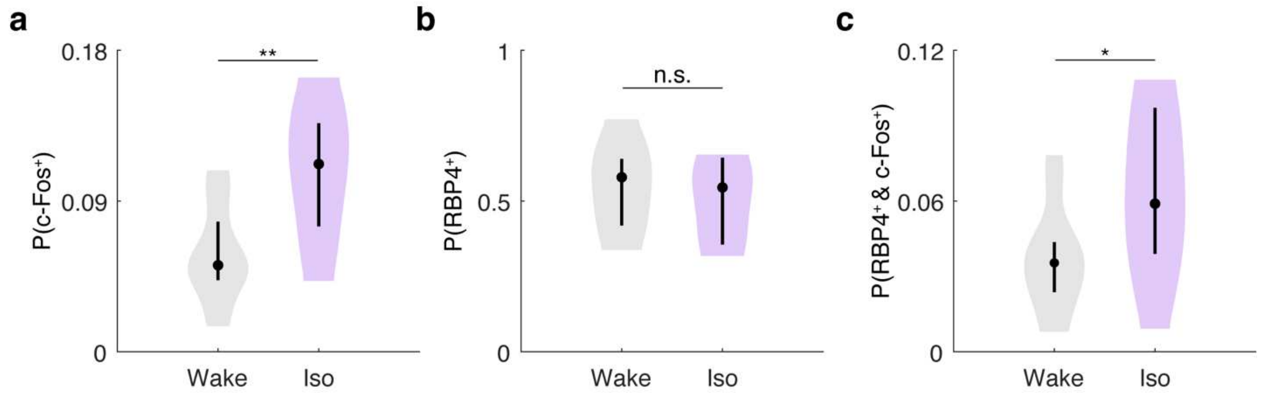

Supplemental Figure 1: PFC Layer 5 neurons are preferentially active under isoflurane compared to wakefulness. RBP4 x Ai6 mice (n=4 per group) were exposed to either 0.9% isoflurane (iso) or 100% oxygen (wake) for 2-hours before rapid sacrifice, brain harvest and immunostaining for c-Fos (activity marker) and NeuN (neuronal marker). Infralimbic, prelimbic, and cingulate cortex were counted separately but grouped together for analysis. **a)** Fraction of NeuN positive PFC cells expressing c-Fos. Mean probability of observing c-Fos expressing neurons across PFC is significantly higher following isoflurane exposure compared to wakefulness (p=0.0021, unpaired t-test). **b)** Fraction of NeuN positive PFC cells expressing RBP4 in PFC. Mean probability of observing RBP4 expressing neurons across PFC was not different across experimental conditions (p=0.5977, unpaired t-test). **c)** Fraction of NeuN positive PFC cells expressing both RBP4 and c-Fos. Mean probability of observing RBP4 and c-Fos colocalized neurons across PFC is significantly higher following isoflurane exposure compared to wakefulness (p=0.0212, unpaired t-test). Data shown as violin plots (black circle shows the median, vertical bar shows interquartile range). Statistical significance denoted by *p<0.05, **p<0.01.

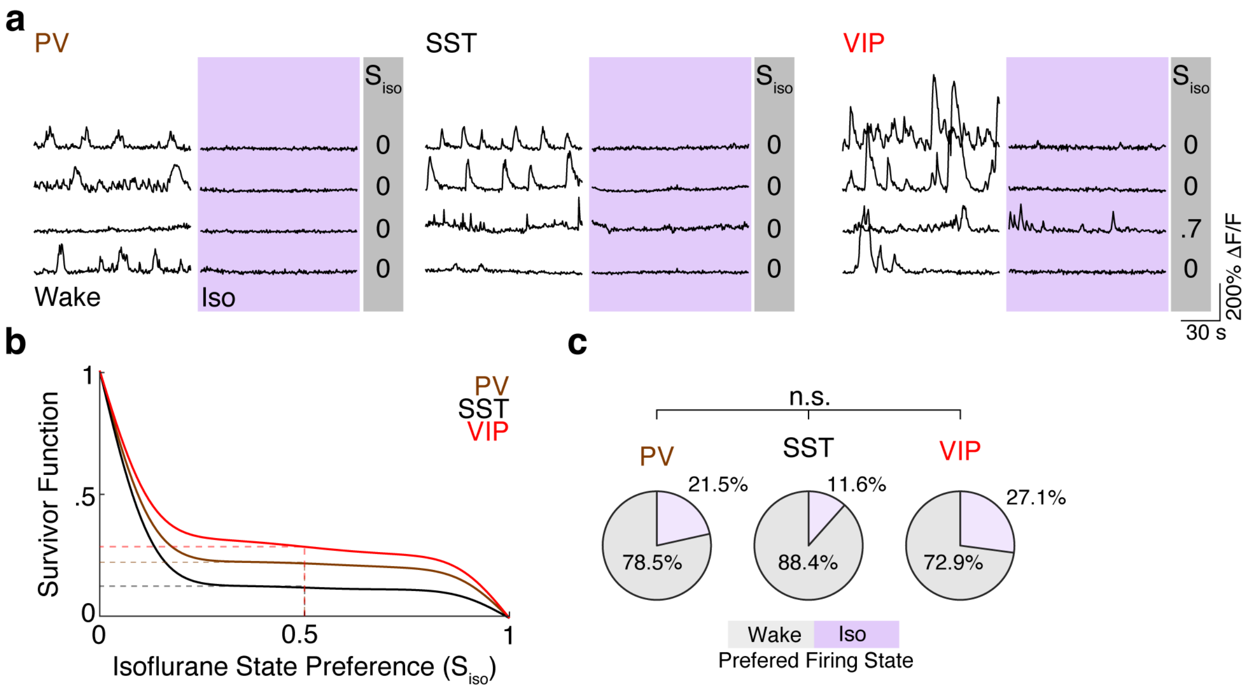

Supplemental Figure 2: Wake-Isoflurane Activity of Prefrontal Cortical Interneurons. a) Representative calcium activity traces from molecularly defined interneuron subtypes shown during wakefulness and under 0.6 % isoflurane (purple): Parvalbumin (PV), somatostatin (SST), and vasoactive intestinal polypeptide (VIP), showing state-dependent activity across wake and isoflurane conditions. Numbers shown at right margin indicate Isoflurane State Preference (S_{iso}) for each neuron (PV n=4 mice, 79 neurons, SST n=3 mice, 69 neurons, VIP n=3 mice, 59 neurons). **b)** Survivor function (P) of S_{iso} across the three neuronal groups in **a.** P(0.5) reflects the fraction of neurons that exhibited higher activity under isoflurane (dashed lines). **c)** Binarized pie charts of preferred activity state. A chi-square test indicates no significant differences in the proportion of interneurons with isoflurane activity preference between groups ($\chi^2(2)=5.062$, $p=0.0796$).

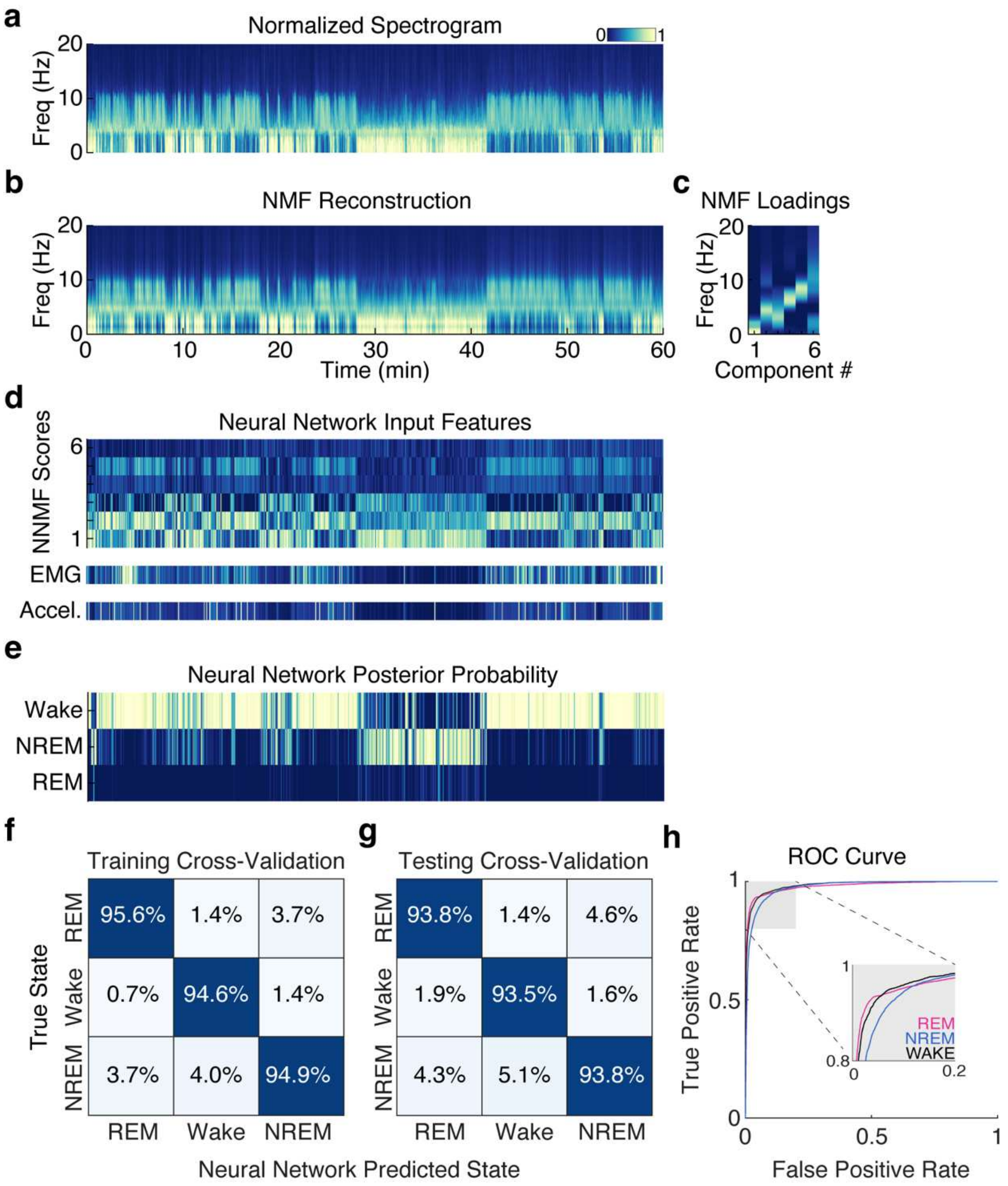

Supplemental Figure 3: Neural network classifier performance for sleep-state prediction using NMF-derived features. a) Normalized EEG spectrogram over a 60 min period used as input for non-negative matrix factorization (NMF). **b)** Reconstructed spectrogram from NMF components. **c)** Frequency loading matrix showing spectral profile of each NMF component. **d)** Corresponding score matrix showing temporal activation of each component across time. The number of NMF components was optimized to explain spectral variance, with 6-components capturing 91.4% of total signal variance. These components, along with synchronized EMG and Accelerometer root-mean-square (RMS) signals were used as input features for neural network classification. **e)** Posterior probability for each predicted vigilance state. The state with the highest posterior probability at each time point was assigned the predicted label. **f-g)** Confusion matrices showing classifier performance during leave one out cross-validation during **f)** training and **g)** testing segments, demonstrating high classification accuracy across all three states. **h)** Receiver operating characteristic (ROC) curves for Wake (black), NREM (blue), and REM (pink) classification, with inset highlighting high-sensitivity region.

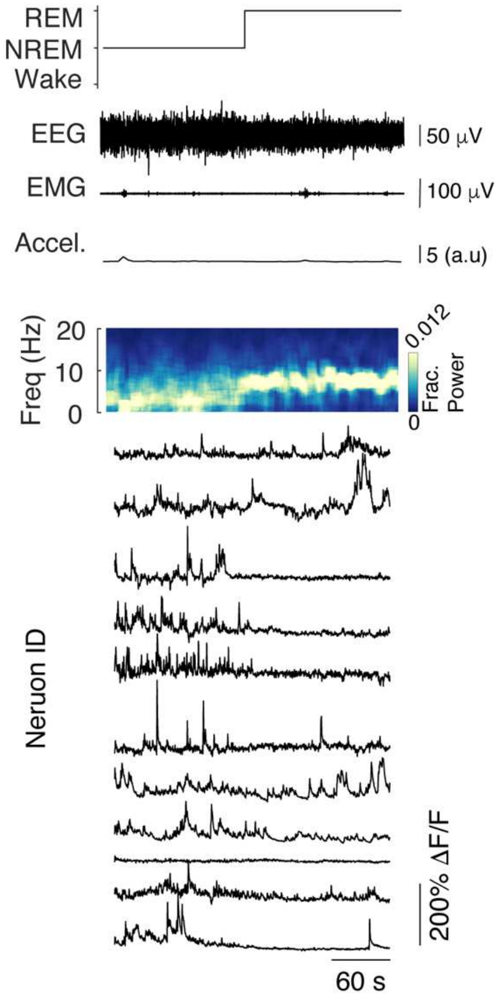

Supplemental Figure 4: Simultaneous EEG, EMG, accelerometer, and calcium activity of NAPs during a NREM-REM transition. 5-minute recording shown. Top to Bottom: Hypnogram, EEG, muscle tone, and movement. Spectrogram is expressed as fraction of power per frequency. Calcium traces from prefrontal NAPs expressing GCaMP following isoTRAP.

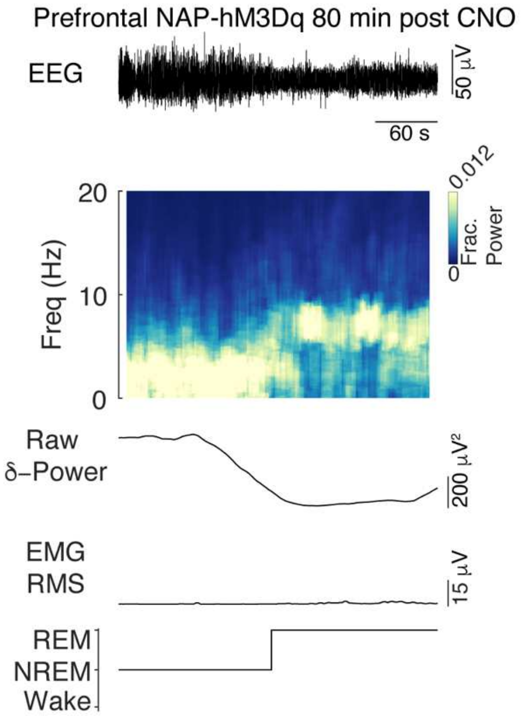

Supplemental Figure 5: Example of NREM-REM transition following chemogenetic activation of NAPs using CNO. 5 min of data shown starting ~80 min following CNO injection in prefrontal NAP-hM3Dq mouse (same as in Figure 2g), illustrating NREM to REM transition. Top panel is EEG trace, second panel is normalized spectrogram, third panel δ-power, forth panel EMG RMS, and bottom panel is hypnogram.

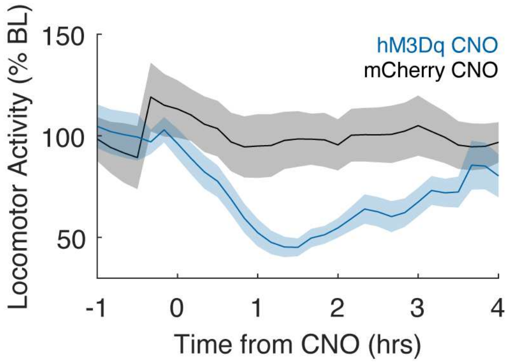

Supplemental Figure 6: Chemogenetic activation of prefrontal NAPs reduces spontaneous locomotor activity. Time course of home cage locomotor activity following CNO injection (t=0) in mice expressing hM3Dq (blue, n=11) or mCherry (black, n=9) in prefrontal NAPs. Activity is normalized to baseline movement 1 hr prior to CNO injection Locomotor activity significantly decreased in hM3Dq, but not in mCherry group. ($F(4,90)=51.43$, $p<0.0001$, 2-way ANOVA, Šídák's multiple comparisons test: mCherry BL vs. 0-1 $p=0.9999$, BL vs. 1-2 $p=0.1638$, BL vs. 2-3 $p=0.9896$, Bl vs. 3-4 $p=0.4927$; hM3Dq BL vs. 0-1, 1-2, 2-3, 3-4, all $p<0.0001$; mCherry vs hM3Dq BL $p=0.2854$, 0-1, 1-2, 2-3, 3-4, all $p<0.0001$). Solid line (shading) represents mean and 95% Jackknife CI.

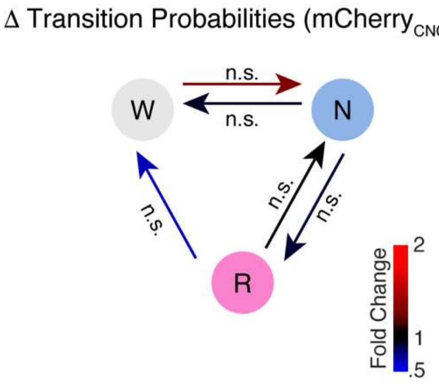

Supplemental Figure 7: Transition Probability Matrix Estimation of Prefrontal NAP-mCherry Mice. Transitions between Wake, NREM, and REM were modeled as a Markov process. Transition probabilities after CNO injection (0.5-2.5 hrs) were normalized by transition probabilities zeitgeber time–matched (ZT 18.5-20.5) period 24 hrs prior to CNO injection. No significant difference was found for control and post CNO period. (n=8 mice, $F(2.599, 18.19) = 0.2672$, $p<.8215$, 2way ANOVA with Geisser-Greenhouse Correction Transition x Time; $F(1,7) =- 13.62$, $p=0.99$, Treatment). Red values indicate a greater transition probability in the CNO condition, whereas blue values indicate a greater probability in the baseline condition.

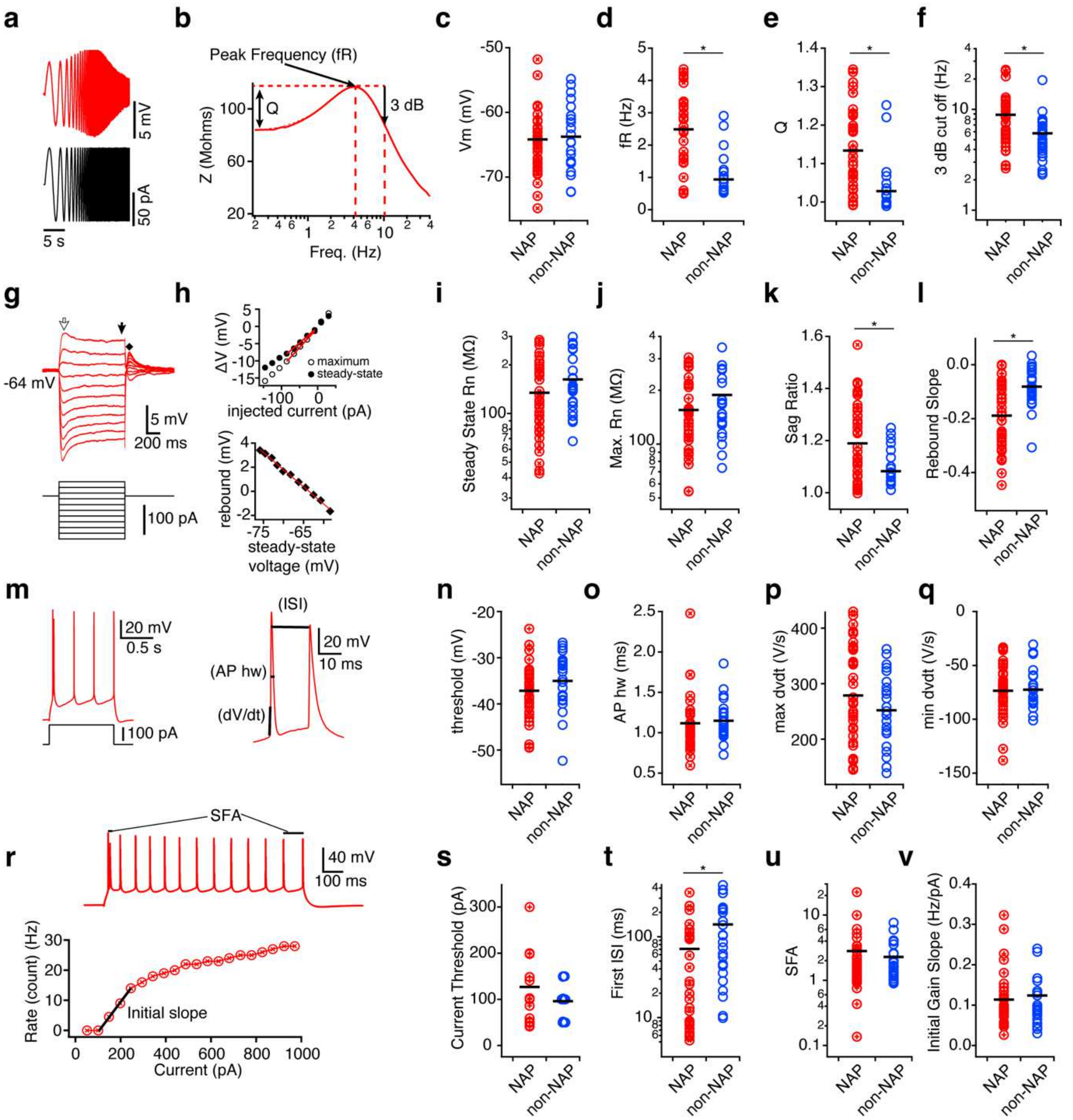
a
5 mV
50 pA
5 s
b
Peak Frequency (fR)
Q
3 dB
Z (Mohms)
Freq. (Hz)
c
Vm (mV)
NAP
non-NAP
d
fR (Hz)
e
Q
f
3 dB cut off (Hz)
g
-64 mV
5 mV
200 ms
100 pA
h
ΔV (mV)
maximum
steady-state
injected current (pA)
rebound (mV)
steady-state
voltage (mV)
i
Steady State Rn (MΩ)
j
Max. Rn (MΩ)
k
Sag Ratio
l
Rebound Slope
m
20 mV
0.5 s
100 pA
(ISI)
20 mV
10 ms
(AP hw)
(dV/dt)
n
threshold (mV)
o
AP hw (ms)
p
max dvdt (V/s)
q
min dvdt (V/s)
r
SFA
40 mV
100 ms
Rate (count) (Hz)
Initial slope
Current (pA)
s
Current Threshold (pA)
t
First ISI (ms)
u
SFA
v
Initial Gain Slope (Hz/pA)

Supplemental Figure 8: Electrophysiological Properties of NAPs. a) Voltage response (upper, red) of NAP to sinusoidal chirp current injection (black): ± 50 pA increasing from 0.2 to 40 Hz. **b)** The calculated impedance amplitude (Z) profile of **a**, with arrows denoting frequency with maximum impedance (resonant frequency, fR), relationship the resonant strength (Q), and the higher frequency at which the peak is diminished by 3 dB. **c-f)** Distribution of resting membrane potential (Vm), fR (U = 206.5, p <0.001, *d* = 1.35), Q (U = 194, p < 0.001, *d* = 1.13), and 3 dB cutoff (U=254, p < 0.001, *d* = 0.71) in NAPs (red circles, n = 37) compared to adjacent non-labeled pyramidal neurons (non-NAPs, blue circles, n = 26). Means are denoted by black bars. **g)** Representative traces of voltage response (red, upper) of NAP to subthreshold current step injections (black, below). **h)** Upper graph is the current-Voltage (I-V) plot of change in voltage in response at maximum (open circle, measurement location is open arrow in **g)** and steady-state (filled circle, measured at filled arrow in **g**). Red lines show linear slopes that are the input resistance (Rn). Lower graph shows for each steady-state voltage potential at the end of the current step, the magnitude of 'rebound' that occurs relative to rest after the current step is turned off. Red line shows slope from which rebound slope is calculated. **i-l)** Distribution of steady state and maximum input resistance, sag ratio (U = 256, p = 0.01, *d* = 0.77) and rebound slope (U = 196, p < 0.001, *d* = 1.06) of NAPs (n = 36) and non-NAPs (n = 22). **m)** Left, representative voltage response of NAP to the first depolarizing step current injection driving action potentials. Right, expanded initial voltage response of cell denoting single spike properties collected. **n-q)** Distribution of voltage threshold, action potential half-width (AP hw), maximum dV/dt, minimum dV/dt of NAPs (n = 34) and non-NAPs (n = 24). **r)** Upper trace, Representative voltage response of NAP to step current injection sufficient to drive at least 10 action potentials. Spike frequency adaptation (SFA) over the course of the step current injection is calculated as the ratio of the first to the last interspike interval (ISI). Lower graph, the number of spikes (plotted as frequency, F) versus injected current (I) of a NAP. **s-v)** Distribution of the minimum current required to drive at least one action potential, the first ISI after the first spike (U = 244, p <0.005, *d* = 1.06), SFA and initial slope of the FI. * Statistical significance denoted by * for samples with P < 0.01 from Wilcoxon rank test, U scores and Cohen's d effect size reported for significant differences in throughout legend.

Supplementary Files

This is a list of supplementary files associated with this preprint. Click to download.

- supfigure1.png
- supfigure2.png
- supfigure3.png
- supfigure4.png
- supfigure5.png
- supfigure6.png
- supfigure7.png
- supfigure8.png
- InputOutputTable.pdf
